# Affine Deformation and Self-Assembly Alignment in Hydrogel Nanocomposites

**DOI:** 10.1021/acs.macromol.3c01638

**Published:** 2023-11-22

**Authors:** Suellen Pereira Espíndola, Ben Norder, Kaspar M. B. Jansen, Jure Zlopasa, Stephen J. Picken

**Affiliations:** † Department of Chemical Engineering, Faculty of Applied Sciences, Delft University of Technology, Van der Maasweg 9, 2629 HZ Delft, The Netherlands; ‡ Department of Sustainable Design Engineering, Industrial Design Engineering, Delft University of Technology, Landbergstraat 15, 2628 CE Delft, The Netherlands; § Department of Biotechnology, Faculty of Applied Sciences, Delft University of Technology, Van der Maasweg 9, 2629 HZ Delft, The Netherlands

## Abstract

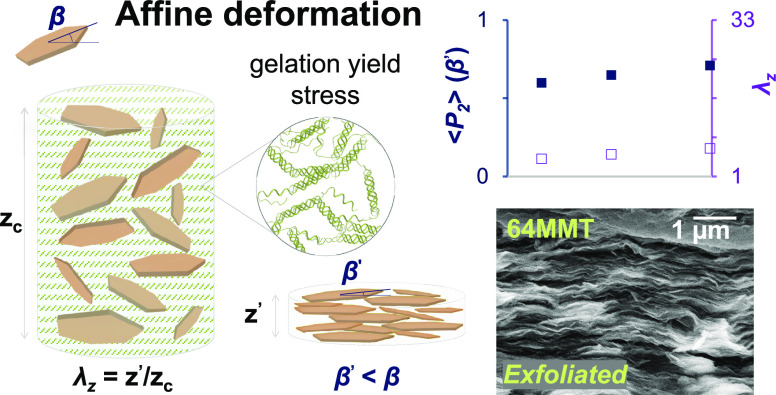

Tailoring the order in hierarchical structures is a key goal of bioinspired nanocomposite design. Recently, nacre-like materials have been developed by solvent evaporation methods that are scalable and attain advanced functionalities. However, understanding the alignment mechanisms of 2D fillers, nanosheets, or platelets remains challenging. This work explores possible pathways for nanocomposite ordering via orientation distribution functions. We demonstrate how the immobilization of 2D materials via (pseudo)network formation is crucial to alignment based on evaporation. We show a modified affine deformation model that describes such evaporative methods. In this, a gel network develops enough yield stress and uniformly deforms as drying proceeds, along with the immobilized particles, causing an in-plane orientation. Herein, we tested the dominance of this approach by using a thermo-reversible gel for rapid montmorillonite (MMT) particle fixation. We researched gelatin/MMT as a model system to investigate the effects of high loadings, orientational order, and aspect ratio. The nacre-like nanocomposites showed a semiconstant order parameter (⟨*P*_2_⟩ ∼ 0.7) over increasing nanofiller content up to 64 vol % filler. This remarkable alignment resulted in continuously improved mechanical and water vapor barrier properties over unusually large filler fractions. Some variations in stiffness and diffusion properties were observed, possibly correlated to the applied drying conditions of the hybrid hydrogels. The affine deformation strategy holds promise for developing next-generation advanced materials with tailored properties even at (very) high filler loadings. Furthermore, a gelling approach offers the advantages of simplicity and versatility in the formulation of the components, which is useful for large-scale fabrication methods.

## Introduction

The precise control and prediction of order in nanocomposite materials remain crucial objectives in the design of advanced nanomaterials. In recent years, materials inspired by the hierarchical structures found in nature have gained substantial popularity.^1−3^ Many methodologies have emerged trying to translate to man-made materials the exceptional property profiles of naturally formed hierarchical structures, such as the ones found in wood, bone, enamel, and nacre.^4−8^ In particular, the thermal, mechanical, and permeability properties of in-plane-oriented nacre-like materials have been intensively investigated.^9^ Nacre is found in the inner shells of mollusks and mussels. Their main feature to be reproduced is the “brick-and-mortar” microstructure, where a high content of mineral aragonite tablets (95 wt %) is decorated by an elastic organic biopolymer. This architecture results in many intriguing properties, from good polymer adhesion to distinct structural colors.^9,10^ In turn, polymer nacre-like materials are lightweight and combine multiple properties, like high stiffness and toughness, via synergistic effects acting between the components of the composite. Good gas barrier properties have also been reported for these composites, which are again linked to the high in-plane orientation of well-distributed platelets.^11,12^ Material optimization has been based on tailoring components, 2D materials, and processes for engineering functional and structural applications (membranes, insulators, electromagnetic shielding material, sensing, biomedical, aerospace devices, etc.).^2,6,13^ However, an in-depth understanding of the possible alignment mechanisms in place would facilitate the successful application of the structure–property relationships found in highly ordered nanocomposites. Especially considering the scalability of the alignment method, it becomes a necessity to better understand the mechanism of alignment.

First, it is important to realize that the 2D fillers in bioinspired composites will have consolidated ordered structures determined by the mobility in the environment in which they are produced. The layered “brick-and-mortar” structures are realized by a *dynamic* interplay of soft and hard phases. Ordered nacre-like materials are usually made from suspension/melt mixing or in situ approaches.^14,15^ For instance, they are produced via casting,^16,17^ vacuum filtration,^18^ and layer-by-layer assembly (L-b-L)^19,20^ methods to achieve high-performance materials.^9^ For some years now, a major development has been the implementation of water-based nanocomposites and large-area fabrication via solvent evaporation, akin to paper-making.^18,21−23^ After that, building block formation, including cross-linking techniques and drying conditions, has been extensively optimized to realize nano- and mesoscopic assemblies of clay silicates (reduced) graphene oxide, MXenes, boron nitride, dichalcogenides, and so forth.^22,24−28^ Most reports follow the rationale that a polymeric core–shell formation and subsequent particle alignment by directed or evaporative self-assembly mechanisms preserve the high aspect ratio of platelets or sheet fillers.^7,8,18^ However, the colloidal ordering principle is seldom explained or explored in any detail. Some point out excluded volume associations,^18,29,30^ as in an Onsager-like treatment.^31^ In any case, better criteria should be used to evaluate the structural ordering, ideally with the evaluation of an orientational distribution function (ODF).^15,24,32,33^

The role of the preferential orientation of 2D materials on the properties of a wide range of composites is well established.^30^ In traditional particle-reinforced polymer composites, orientational order is usually studied by fitting a distribution function over experimental structural data such as crystallite diffraction patterns. Unfortunately, unlike in nacre mimetics, this essentially covers composites with low filler loading^10^—up to 5 vol %. This work focuses on a new perspective and strategy for the structural ordering of lamellar nanocomposites up to high loadings. We consider a common waterborne system in which an entangled network or hydrogel formation rapidly restricts 2D nanofiller mobility. In particular, the effect of a frozen-in environment on composite alignment is evaluated. The ordered nanostructures are tested over a wide range of 2D nanofiller fractions, surpassing the polymer content and going into the highly confined polymer regime. Since we aim to understand the underlying dynamics of alignment in nacre-like lamellar systems, we scrutinize the consistency of an ODF, the modified affine deformation model, to describe each produced hydrogel system. This is of special importance in identifying the triggers for structural ordering and associated mechanical and transport property enhancement. Despite the focus on 2D nanomaterials and nacre-inspired research, we expect this to extend to other types of grain morphologies (tubes, rods, and short fibers) and other solvent systems. Our theoretical perspective on nanoparticle movement restriction in waterborne methods should also be relevant to industrial scalability. In general, a network-based orientation should allow for less energy- and time-intensive methods. More importantly, meticulous control of the preferred hierarchical ordering is crucial to the development of consistent large-area and large-volume manufacturing.

In summary, our study attempts to shed light on the alignment mechanism of nacre-like materials and provide insight into the design and optimization of high-load advanced nanomaterials with improved properties.

## Orientation Model

Several high aspect ratio 2D nanofillers can reinforce and functionalize composite materials with “brick-and-mortar” structures. Even though numerous studies have proven that highly anisotropic nacre-inspired structures develop from initially isotropic hydrocolloidal suspensions, the exact orientation mechanism is often unsolved. Here, we will first explore the assumptions for a certain nanostructuration and possible alignment mechanisms (self-assembly vs affine deformation). Next, we apply this to a hydrogel model system that should be ruled by affine deformation. In this work, we research the influence on properties of parameters that are crucial to structural ordering, that is, the 2D nanomaterial concentration, aspect ratio, and average order parameter, for example, ⟨*P*_2_⟩. The variables involved are extensively explored to establish how they influence the anisotropic mechanical stiffness and gas permeability properties of the nanocomposite. The main goal is to test the affine deformation alignment strategy up to very high loadings using facile fabrication methods.

### 2D Nanocomposite Alignment

**Figure 1:**
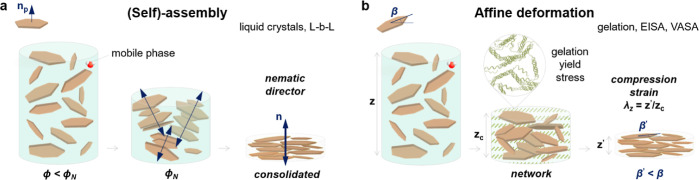
Schematic illustration of the nacre-inspired nanocomposite preparation via (self)-assembly methods (a), such as liquid crystals and L-b-L, or hydrogel affine deformation methods (b), such as gelation, EISA, and VASA. In assembly mechanisms (a), starting at the critical concentration, ϕ_N_, a preferred orientation of particles with respect to the film plane develops and the nematic-like uniaxially aligned structure is consolidated by solvent removal. In affine deformation (b), yield stress builds from network formation or (pseudo)gelation and immobilizes the 2D particles until the composite is fully immobilized by solvent evaporation (critical concentration represented by gel height *z*_c_). The vertical strain, λ_*z*_, is the factor that induces the orientational order in (b). This strain is assumed to result in an affine compression, i.e., the local deformation is equal to the global deformation. The average angle between platelet and film normal, β, decreases with particle alignment by either excluded volume (a) or shrinkage (b) effects.

Figure 1 classifies current waterborne nacre-like composite fabrication methods as mechanisms driven by self-assembly or affine deformation. Many nacre-inspired reports have focused on optimizing precursors and conditions via L-b-L deposition or a so-called self-assembly mechanism. Nevertheless, the required conditions for the formation of 2D material assemblies must be contemplated. In systems where the order is formed through assembly, an externally induced or spontaneous process causes the randomly oriented 2D materials to align along a preferred axis or director. For instance, a nematic environment can develop by applying an external field or through excluded volume interactions, which depend on a high particle concentration and aspect ratio (Figure 1a). The already aligned system is then consolidated by an external field, dense network formation, and/or drying. Only when this happens spontaneously, normally through localized interactions in a mobile phase, should the mechanism be termed self-assembly. Typical examples are thermotropic or lyotropic liquid crystal formation.^34^ In such composites, the ODF is usually quite well described by a mean-field theory, that is, either the Maier–Saupe or the Onsager model.^31,35,36^ Theoretically, Onsager-like nematic phases could develop from repulsion between the hard-core nanoparticles. However, it is fair to assume that most accounts on nacre-like materials do not exceed the required critical particle concentration. Usually, the lyotropic particles described by Onsager’s formula have a monodisperse character, a trait not so easily found in the employed 2D nanoparticles. On the other hand, the Maier–Saupe model (eq 1) was initially proposed based on soft-core attraction forces. The theory assumes a molecular or particle long-range distribution that determines a nematic mean-field potential or influences the basal angle of an individualized particle. The Maier–Saupe model might be considered for the specific cases of an externally directed flow (e.g., electromagnetic polarization) or a very controlled L-b-L deposition. Nevertheless, both Onsager and Maier–Saupe ODF functions will adopt a Gaussian shape at a high level of orientational order (high ⟨*P*_2_⟩).^37^ A point often overlooked is that the nanoparticles can frequently get immobilized in a hydrogel or entangled network, in which case one must exclude the possibility of them further aligning via mean-field spontaneous self-assembly.

We have previously implemented an alternative theoretical framework to waterborne nacre-like nanocomposites, for example, based on nanoclay, graphene, or calcite.^15,24,32^ The modified affine deformation model (eq 2) describes an orientation mechanism in which a (pseudo)network is formed in the matrix phase, developing enough yield stress to immobilize the nanoparticles while drying (Figure 1b). Here, the word affine infers that the local deformation is the same as the deformation in the global system, a term adapted from studies of the junction networks in ideal rubbers (Kuhn and Grun, 1942^38^). A uniform gelling matrix shrinkage drives the in-plane alignment to the entrapped particles. More specifically, the structural ordering is controlled by the drying-induced vertical strain (λ_*z*_) experienced by the network. One advantage of such a mechanism comes from nanoparticle immobilization with the aid of an appropriate matrix solvent, thus initially preventing loss of filler aspect ratio. This translates to nanostructures that are effectively exfoliated/intercalated and well-aligned,^39^ which is crucial for final material properties, for example, gas transport and mechanical properties. Even though this mechanism serves as a separate nacre-inspired strategy, many studies have resulted in a similar composite preparation method via trial and error. Those include gelation mechanisms and evaporation-induced self-assembly (EISA) or vacuum-assisted self-assembly (VASA).^8,26,40,41^ To name a few, Wu et al. (2014)^23^ used an in situ hydrogelation mechanism to lock magnetically aligned graphene oxide platelets. The orientation mechanism described resulted in functionalized soft materials with good order parameter values (⟨*P*_2_⟩_Maier–Saupe_ = 0.8). Zheng et al. (2020)^42^ also reported high-performance biocomposites from chitosan/nanoclay cross-linked by either glutaraldehyde or Pd^2+^ cations in water. Thus, cross-linking agents should also result in the affine deformation of network-embodied nanoplatelets. Recently, Zhou et al. (2022)^25^ combined an oil-spreading shear-flow alignment of alginate/MXene with divalent ion-induced cross-linking. This fast hydrogelation efficiently immobilized the aligned 2D filler in the matrix before dry-settling, resulting in outstanding mechanical properties (elastic modulus of 60 GPa). As shown above, the affine deformation mechanism can be an effective pathway to attain very highly oriented structures in nanocomposites but has mostly been explored implicitly.

## Results and Discussion

### Characterization of Hydrogel-Based Nanocomposites

According to this concept, our experimental design starts with selecting a compatible and versatile nacre-like hydrogel system. Due to the thermal transitions in gelatin (type A) and the high aspect ratio of montmorillonite (Na-MMT), we have chosen the well-miscible gelatin/MMT composites to study this theory. In conjunction with that, we have explored the possibility of high loadings, going as far as 80 wt % MMT composite fraction. We can control the mobility and total strain applied on both nanoplatelets and the gelling matrix during film casting through simple solvent evaporation. In theory, we test the effect of a (uniform) network deformation using a thermo-reversible gel for rapid 2D particle immobilization as the exfoliated suspension cools to room temperature.

**Figure 2:**
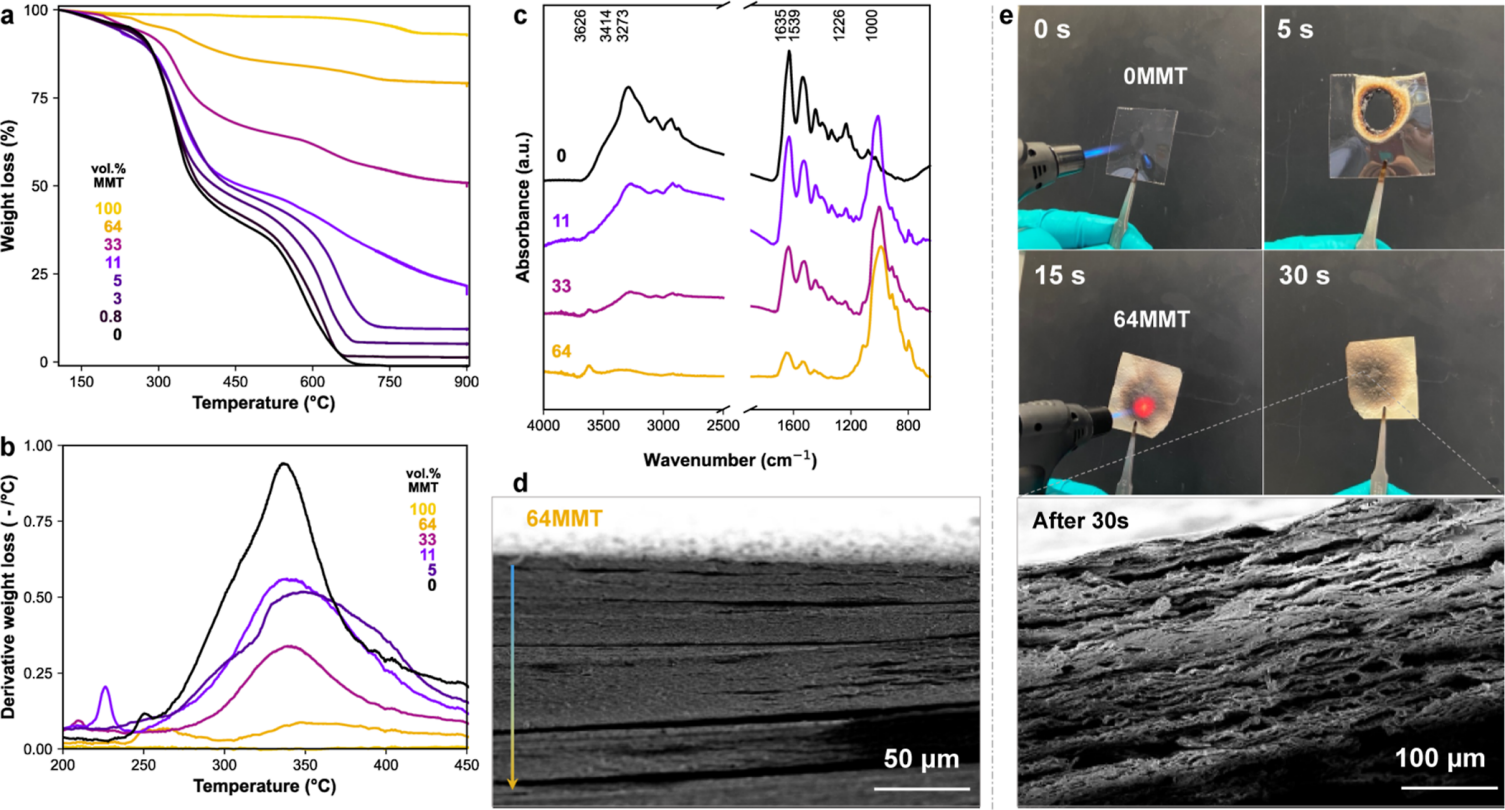
Characterization of nacre-inspired nanocomposites of gelatin/MMT cast from 3 wt % suspensions. (a) TGA scan of neat gelatin type A (black), clay-based composites up to high volume fraction (64 vol %), and pure clay (bright yellow). (b) Respective DTG for the same film materials at the gelatin oxidative pyrolysis interval. (c) FTIR spectra of dried neat gelatin type A (black) and clay-based composites. (d) SEM cross-section image of gelatin/MMT composite with high loading shows homogeneous lamellar structure without clay aggregates (arrow indicates the film drying direction). (e) Oxidizing flame test of the gelling matrix and 64 vol % MMT composite (thickness 0.1 mm), including cross-sectional SEM after burning.

The gelatin/MMT nanocomposite films were easily fabricated under ambient conditions. The exact MMT composition content was confirmed by thermogravimetric analysis (TGA, Figures 2a and S2), and the MMT volume fraction *X* is herein abbreviated as *X*MMT. Thermal stability and degradation behavior were observed under an oxidative atmosphere. Figure 2b shows composites’ thermal mass loss derivative curves, DTG, close to the gelatin oxidative pyrolysis interval. We observe the main degradation of gelatin around 340 °C, in which the DTG intensity represents the degradation rate. We note that the DTG intensity decreased with increasing MMT loadings. This is likely due to the lower polymer mass; however, gas entrapment is also expected.

**Figure 3:**
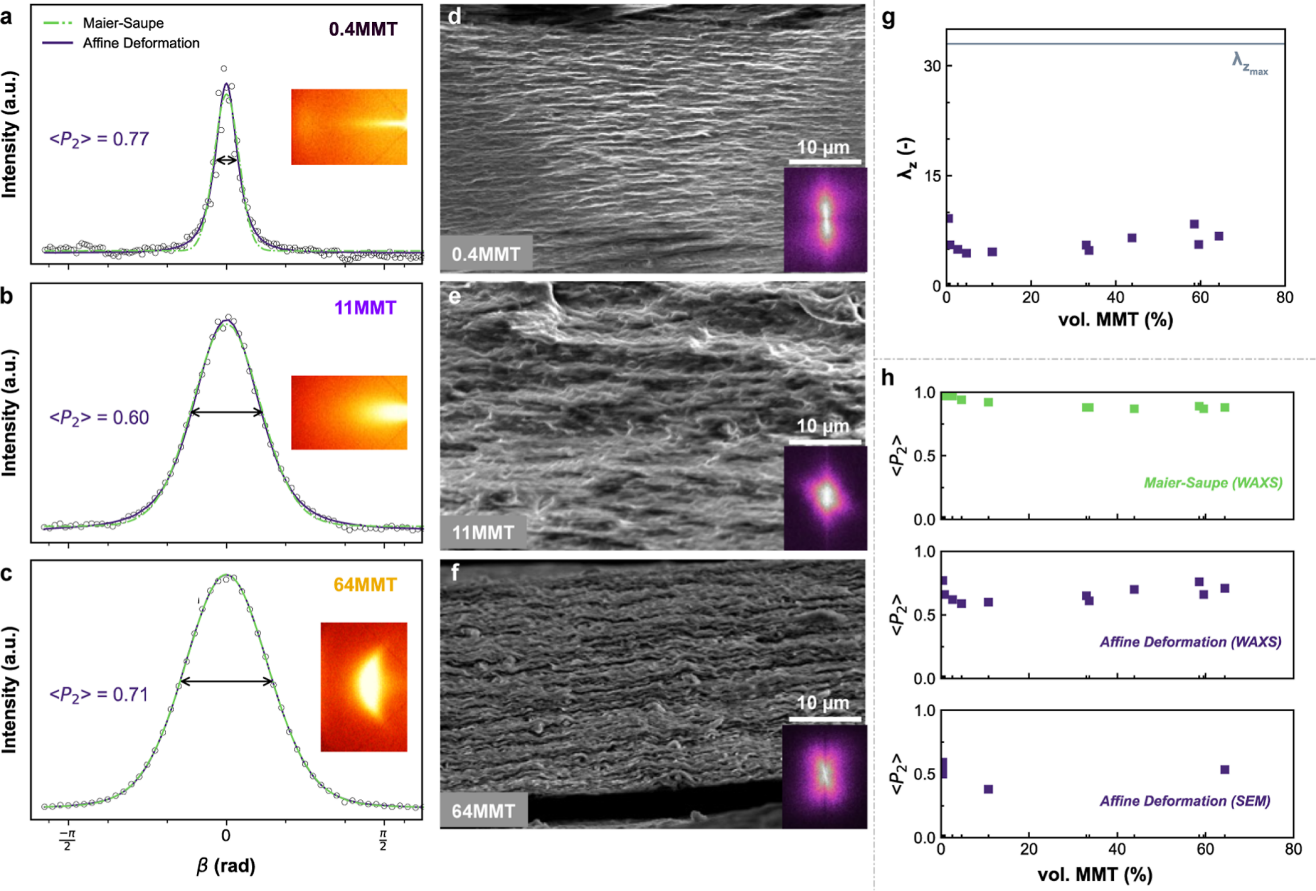
WAXS showing 001 Bragg reflections, azimuthal intensity profiles of the primary diffraction peak, calculated orientation distribution functions of affine deformation (dark purple solid lines), and Maier–Saupe (light green dashed lines) for nacre-inspired nanocomposites 0.4MMT (a), 11MMT (b), and 64MMT (c). The ⟨*P*_2_⟩ values depicted in the images were determined via affine deformation ODF. SEM cross-section image of nanocomposites 0.4MMT (d), 11MMT (e), and 64MMT (f), where insets show corresponding FFT of the images for peak integration. (g) Degree of compression, λ_*z*_, obtained via affine deformation fits (WAXS). (h) Order parameter ⟨*P*_2_⟩ values estimated from the orientation distribution functions of Maier–Saupe on WAXS data (top), affine deformation on WAXS data (middle), and affine deformation on SEM data (bottom).

In Figure 2c, the Fourier-transform infrared spectroscopy (FTIR) spectra are shown for the gelatin and gelatin/MMT composite dry films. We can observe in the gelatin amide region (1800 to 1000 cm^–1^) an overall reduction of amide absorption bands (C=O stretching at 1635 cm^–1^, N–H deformation and C–N stretching at 1539 and 1226 cm^–1^) with increasing nanoclay fractions. This might indicate strong protein-nanoclay associations, imparting changes in the gelatin triple helix supramolecular structures. A relatively steady increase in the intensity of the inorganic MMT peak is also observed around 1000 cm^–1^ (stretching of Si–O and Al–O). Up to high loadings, 64 vol % MMT, we observe no aggregation phenomena, and a uniform dense lamellar structure is observed via cross-sectional scanning electron microscopy (SEM) (Figures 2d and 3d–f). In fact, the composites show a nacre-like structure in which MMT platelets are oriented perpendicular to the direction of hydrogel drying. In addition, our previous wide-angle X-ray scattering (WAXS) and focused ion beam scanning electron microscopy (FIB-SEM) analyses show that the polymer is homogeneously distributed within the lamellae, resulting in exfoliated nanostructures.^39^

A flame resistance test was performed for a few nanocomposites after 50% RH equilibration; see Figures 2e and S12. This test provides a rapid way to assess nanocomposite flame retardancy. The unfilled gelatin A film instantaneously melts and produces smoke. On the other hand, as expected from clay-based materials, the composite with very high filler loading keeps its integrity even after very high-temperature exposure (∼1000–1970 °C) while not producing smoke. The flame exposure causes a spot of blackbody radiation glow from the front side (red spot), while that is almost not detected from the rear side, indicating an enormous thermal gradient in the sample on the order of 3000 K/mm (Figure S12). We also note that the gelatin/MMT nanocomposites possess beneficial intumescent behavior, as supported by the SEM of the char, where the film expands to a mesoporous structure but sinters to a structurally similar (lamellae) material. The films were around 0.1–0.2 mm thick, and the volumetric expansion is related to the localized formation of CO_2_, CO, and H_2_O gases from the organic fraction decomposition. The nonflammability and heat-shield behavior presumably must come from a high level of orientation in the system at high loading, which diminishes the oxygen and low molar mass volatile component’s diffusion through the films.

### Origin of High Orientational Order

An orientation distribution function is a suitable tool to investigate which alignment mechanism is in place. In natural clay-rich materials, like shale rock or soil crust, ODFs are also fitted to X-ray scattering data to obtain the degree of “single platelet” orientation.^33^ Clay system coordinates are usually defined by a frame coinciding with the axis of symmetry (i.e., the *z*-axis in Figure 1). The orientation distribution function describes the probability density that a particle is found between stepwise-changing orientations, defined over the platelet basal angle (i.e., β angle in Figure 1). The ODF applied to this angular framing is analyzed as an infinite series of Legendre’s polynomial functions or momenta. It is common to limit the extent of orientation analysis to the second moment, ⟨*P*_2_⟩ (eq 3), also known as the “Hermans orientation factor f” or “the order parameter S”. For a perfectly aligned material, it should be ⟨*P*_2_⟩ = 1, and for a perfectly isotropic material, ⟨*P*_2_⟩ = 0.

From the preferred orientation of the MMT nanoplatelets, the X-ray [001] reflection can be evaluated as a function of the azimuthal angle. The development of [001] features in WAXS analysis at a glancing angle of the film surface, perpendicular to the drying direction, confirms the orientational order at the nanoscale (Supporting Information, Text S1 and Figure S3). When the analysis is performed perpendicular to the film surface and parallel to the drying direction, we observe an absence of features, indicating axial symmetry (Figure S3). The integrated azimuthal profiles at the highest intensity are fitted to the Maier–Saupe and affine deformation of the ODFs (Figure 3a–c). Due to X-ray instrument broadening effects, we fitted the affine deformation convoluted to a Gaussian function (Supporting Information, Text S1 and Figure S5). The modified affine deformation model well describes the azimuthal intensity profiles of the films (Efron’s pseudo-*R*^2^ values >0.96 and lower chi-squared statistics, Table 1). These results confirm that the anisotropic films result from affine compression during the fibrous network drying phase. Curiously, the neat gelatin also showed a highly layered and smooth microstructure, typical of a brittle material fracture^43^ (Figure S4). The gelatin matrix showed a degree of ordering resulting from aggregates of renatured triple-helices.^44^ In addition, fast Fourier transforms (FFTs) of the SEM images also confirmed hierarchical anisotropy in the composites, showing a high in-plane microscale orientation (Figure 3d–f, insets).

**Table 1:** Obtained Parameters from Orientation Distribution Functions Applied to Gelatin/MMT Azimuthal Profiles and the Effect of Final Sample Geometry on Strain^a^

		Maier–Saupe			affine deformation (Gaussian convoluted)				
sample	measured radial strain (λ_*r*_^2^)	α	RSS	Efron’s pseudo-*R*^2^	vertical strain (λ_*z*_)	critical total solids (100/λ_*z*_, wt %)	RSS	Efron’s pseudo-*R*^2^	ideal uniaxial strain (λ_uniaxial_ = λ_z_*λ_*r*_^2^)
0.4MMT	1	75.7	0.308	0.946	9.2	11	0.213	0.963	9.3
11MMT	2.2	10.2	0.015	0.997	4.6	22	0.008	0.998	10.1
33MMT	1.3	7.0	0.005	0.9996	5.5	18	0.004	0.9996	7.2
64MMT	1.3	6.7	0.003	0.9998	6.8	15	0.002	0.9998	9.1

a λ_*r*_^2^: degree of measured area shrinkage or strain in radial direction; α: fwhm parameter in Maier–Saupe; λ_*z*_: vertical strain in *z*-direction calculated from fwhm parameter in affine deformation; λ_uniaxial_: expected ideal uniaxial strain via volumetric correction of an incompressible rubber (λ_*x*_ λ_*y*_ λ_*z*_ = λ_*r*_ λ_*r*_ λ_*z*_ = 1); RSS: residual sum of squares; and Efron’s pseudo-*R*^2^: fit probabilistic pseudo-*R*^2^.

Table 1 shows the full-width at half maximum (fwhm) coefficients of orientation functions, which are directly related to the order parameter. In the affine deformation, the fitted variable λ_*z*_ represents an equivalent vertical compressive strain. By inverting the value of λ_*z*_, we estimated the critical total solid concentration needed for the film-forming suspension to achieve particle immobilization. Generally, the nanocomposites have already developed yield stress in the 22 to 11% solid regime. By comparison, intercalated alginate/MMT composites previously showed a critical concentration range for immobilization between 60 and 28% solids for samples with 5–50 wt % MMT, respectively.^32^ The gelatin/MMT composites were initially cast at 3% solids. If the yield stress had developed immediately upon casting, the maximum degree of vertical compression should have been 33 (λ_*z*__max_). The lower experimental λ_*z*_ values indicate that the hybrid gels were not completely affinely deformed from the outset. A degree of nonaffinity is inevitably present in hydrogels and arises from a fibrous filament bending and/or micro inhomogeneities in a determined sample.^45^ A variation in gel dynamics was also observed, with the macroscopic gels formed above 11 vol % MMT seeming to consist of fragmented networks.^39^ In addition, these MMT concentrations led to gels that contracted and displayed a lower adhesion to the substrate during drying, causing lateral shrinkage (Figure S6). The presence of a radial strain, λ_*r*_, obviously lowered the amplitude of the uniaxial compression, λ_*z*_, and the resulting anisotropy (Table 1). By analogy, the MMT particles served as tracer particles to the hydrogel affine deformation.

The ⟨*P*_2_⟩ order parameter was calculated from the fit parameters extracted from Maier–Saupe (over WAXS) or affine deformation models (over WAXS and SEM) (Figure 3h). From WAXS, the ⟨*P*_2_⟩_Maier–Saupe_ values ranged from 0.87 to 0.99 and the ⟨*P*_2_⟩_affine_ values ranged from 0.59 to 0.77. These values are well above the ones found for most oriented composites. Note that, from the theoretical formalism, ⟨*P*_2_⟩_affine_ values always will be lower than ⟨*P*_2_⟩_Maier–Saupe_. The high order parameter found for filler fractions up to 5MMT may be linked to unresolved WAXS structures (streak features are unresolved a priori; see Supporting Information, Text S1), which gives uncertainty in ⟨*P*_2_⟩ determination. In general, the absolute values for each nanocomposite are more difficult to compare due to instrument broadening, radial film shrinkage (*xy*-plane), and the influence of high MMT fractions on the smoothness of the particles. Nevertheless, ⟨*P*_2_⟩_affine_ estimated from the layered microstructures follows a trend with nanoclay concentration similar to that for the values calculated from diffraction data. This is clear evidence that the hydrogel compression history is the dominant factor. The overall trend of ⟨*P*_2_⟩_affine_ up to high MMT loadings (33 to 64MMT) indicates that the degree of orientation was at least maintained (no significant differences can be claimed, *p* > 0.05). This semiconstant affine order parameter oscillated around the high value of 0.7.

Notably, ordering can be found at the global mesoscales and microscales and simultaneously at the nanoscale. These two levels of ordering are entirely independent. Hence, the ODFs can also show a convoluted signal from an Onsager-like nanoscale orientation (from an excluded volume). In our system, it is plausible to interpret this as the cause of the change in ODF shape at elevated 2D filler loadings (Figure S5). The Onsager-like orientation mechanism should yield a Gaussian-like function, which is indeed observed in azimuthal profiles above 33MMT fractions. In this case, excluded volume interactions inflicted nanoscale order in the cases of concentrated 2D nanomaterial. This could be linked to the apparently increasing trend in ⟨*P*_2_⟩_affine_ in the high filler region. This observation is made in analogy to the orientation theory commonly applied to nematic liquid crystals. The director field describes the long-range orientation (microscale), whereas, at the molecular director level, the structures can be random or oriented from (flow-induced) orientation correlation.^46^ Thus, for instance, a relatively low level of alignment via affine deformation is possible while locally very highly ordered nanosized “truncated” domains are formed. This result was highly unexpected since the hydrogel network should limit nanoparticle mobility, thereby preventing structures to form. Thus, it makes sense to assume that excluded volume interactions are acting at the nanoscale, which ultimately results in considerable regularity in the particle spacing.

### Mechanical Structure–Properties

**Figure 4:**
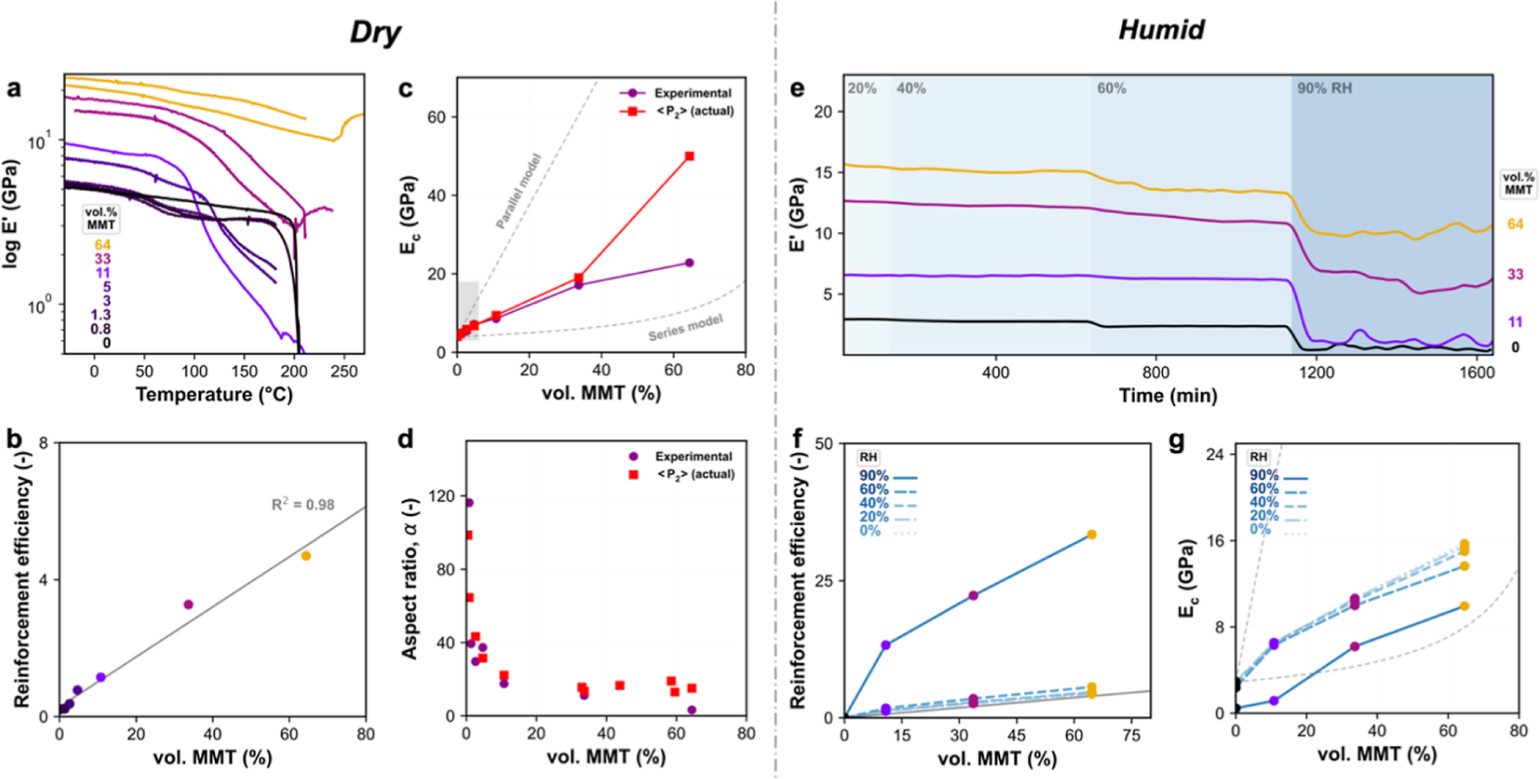
Mechanical properties of nacre-inspired nanocomposites with varying nanoclay loading acclimated to dry or humid conditions. (a) DMTA of gelatin and gelatin/MMT nanocomposites. (b) Reinforcement efficiency based on dry storage modulus ((*E*_c_ – *E*_m_)/*E*_m_) for a wide range of MMT loading. (c) Dry storage modulus via experiments and ⟨*P*_2_⟩-based calculations. Boundaries (dashed lines) are set by Halpin–Tsai in parallel or series models. Gray rectangle is a guide for a typical concentration range of MMT in conventional composites. (d) Back-calculated effective aspect ratio, α, of the MMT using Halpin–Tsai (eq 4) for the various MMT loadings. (e) DMA of gelatin and gelatin/MMT nanocomposites at 30 °C and varying RH intervals. (f) Reinforcement efficiency based on humid storage modulus ((*E*_c_ – *E*_m_)/*E*_m_) for a wide range of MMT loading. (g) Humid storage modulus via DMA/RH experiments. Boundaries (dashed lines) are set by Halpin–Tsai in parallel or series models.

The gelatin system allowed us to push the system boundaries in MMT loading with highly oriented samples, even at 64 vol % filler. Similar composites fabricated with synthetic polymers traditionally go to low volume fractions, around 0.5–5 vol %, to prevent 2D particle aggregation.^14,47,48^ Only recently, solvent-cast composites with higher loadings, roughly above 30 vol %, have been presented.^21,22,49^ From Figure 4, it is evident that the mechanical properties of films are positively affected by an increasing fraction of MMT platelets. In Figure 4a, a general increase in thermal stability with the MMT fraction is also observed, probably a sign of decreased chain mobility. In addition, the highest fraction, 64MMT, shows clear sintering and a high modulus at elevated temperatures (above 240 °C). Probably, the samples went through complete dehydration (denaturation) and browning reactions in the low-oxygen atmosphere of testing. The enhanced thermomechanical properties are attributed to a synergetic effect of the highly ordered nacre-like structure and the strong electrostatic interactions between MMT and gelatin matrix.^44,50^ However, the highly ordered alignment of nanoplatelets is the main factor ruling their good mechanical properties, even at elevated humidity (Figure 4a,e). We observed a monotonic dependence of dry elastic modulus with clay content all the way to 64 vol % MMT. We conclude this from the almost perfectly linear reinforcement efficiency of the matrix in the dry state (Figure 4b). This exceptional trend in the reinforcement was kept even for the higher ranges of relative humidity (RH) (up to 60% RH; Figure 4f). In a saturated water environment, the modulus of the gelatin matrix changed dramatically due to solvent plasticization (Figure 4e), while this effect is much less pronounced for the highly loaded composites, which maintained their mechanical stiffness (Figure 4f).

### Mechanical Aspect Ratio

The mechanical data are better understood by using the rationale of Halpin–Tsai’s composite theory. The Halpin–Tsai model, eq 4, converges into a series or parallel model by changing the filler shape factor, which depends on geometry, orientation, and aspect ratio. The model converges to a series form if the shape factor is much lower than the modulus fraction composite/matrix due to a low aspect ratio (eq 5). If the aspect ratio is high, then the model converges to a parallel rule-of-mixing type of reinforcement (eq 6). In Figure 4c, we plot the nanocomposite modulus within the Halpin–Tsai boundaries. The moduli of dry specimens from experimental and order-parameter-corrected (eq 7) methods are depicted together. We observed a continuous increase in composite modulus with filler volume fraction. Although, at high loadings, there was a disparity between experimental and actual ⟨*P*_2_⟩_affine_-based values. This cannot be attributed to a lowered aspect ratio due to particle stacking, which would have been present in WAXS and SEM data.^39^ Since all the composites were exfoliated, the main contribution to the modulus should have been the orientational order.

In Figure 4d, we monitor the evolution of the aspect ratio with particle concentration, back-calculated from Halpin–Tsai (eq 4). We have found at least a 2-fold decrease in the *effective* aspect ratio, initially predicted to be ∼200 nm. Some decrease was expected since ⟨*P*_2_⟩ is lower than 1. However, this significant decay might be an artifact of gelatin-specific binding and indirect MMT–MMT interactions such as wrinkling phenomena. Hence, α could be interpreted as from not the entire particle but the averaged rigid parts of a nanoplatelet. In accordance with that, we observed structural undulations due to MMT waviness from multiple data (e.g., Figure 3f). The Halpin–Tsai equation is based on the filler being monodisperse and totally rigid, for example, ceramic discs, which is typically demonstrated for low-volume fractions. Thus, based on a degree of 2D material waviness, the real *E*_f_ could decay by at least a factor of 2 or more. This has been observed before in fiber reinforcement, for example, in aramid fibers, *E*_f_ goes range from 240 to ∼80 GPa due to the pleated sheet structure, even when there is a high ⟨*P*_2_⟩.^51,52^ Therefore, particularly at low *d*-spacings, such as from 33MMT,^39^ the Halpin–Tsai model couples the effects of high filler concentrations with aspect ratios.

Recent studies suggest the importance of absolute particle size on polymer gel mechanics,^53−55^ which is not included in the Halpin–Tsai model (eq 4) where the matrix properties are considered to be invariant. We interpret this size effect to be primarily changing the polymer dynamics at the polymer–particle interface, which is not relevant for the far-below *T*_g_ solid-state mechanics discussed here. Modification of the polymer–particle interface dynamics should, however, be relevant for the nanocomposite permeability, as discussed later.

### Transport Structure–Properties

**Figure 5:**
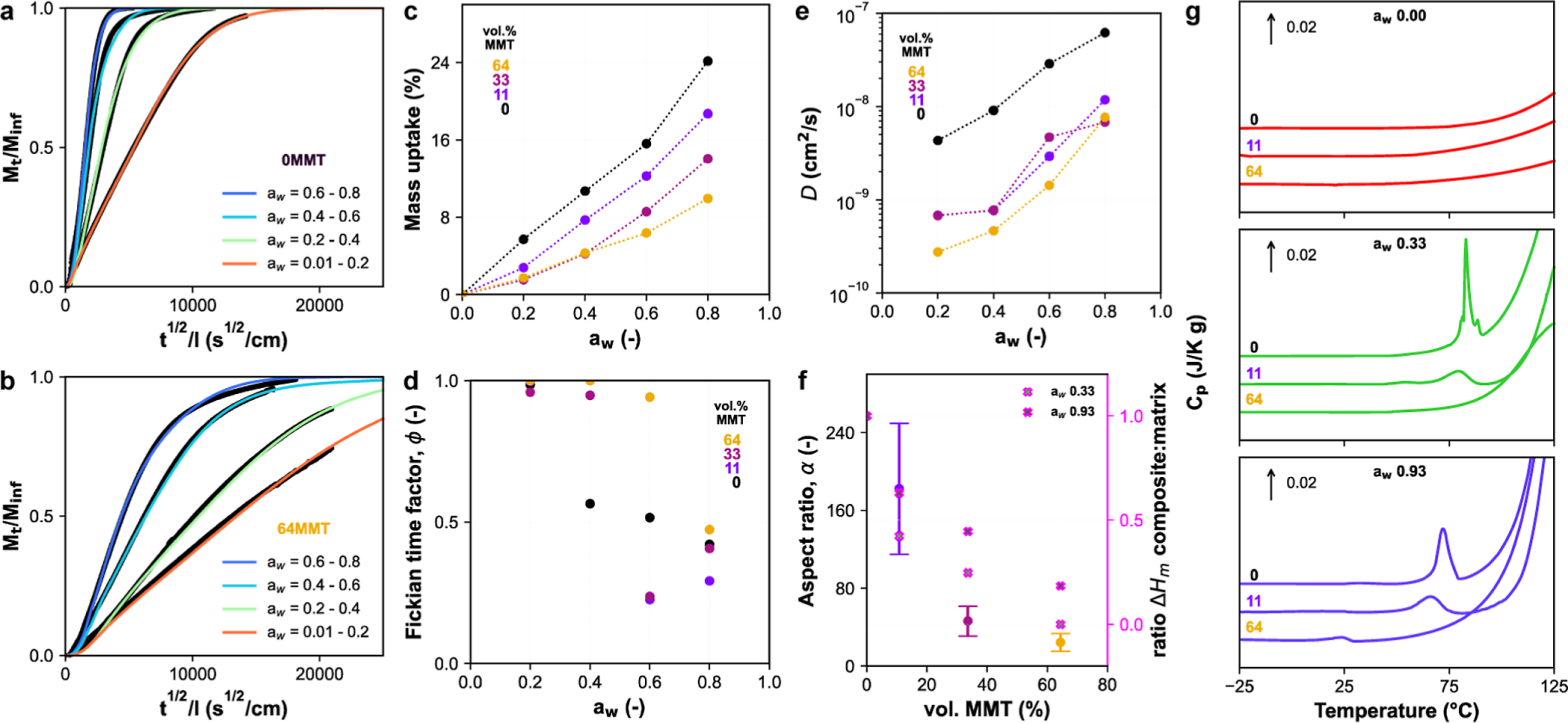
Kinetic water sorption at different water activities of (a) gelatin and (b) 64MMT nanocomposite at 30 °C. (c) Equilibrium water vapor sorption isotherms of matrix and nanocomposites. (d) Fickian time factor, ϕ_F_, a weighing parameter for the extent of Fickian type of diffusion in eq 9a, for matrix and nanocomposites. (e) Water diffusion coefficient, *D*, calculated with eq 9b from the kinetic sorption curves of matrix and nanocomposites. (f) Aspect ratio, α, of the MMT back-calculated from *D* for various MMT loadings, where the reported value is an average of the studied water activities (*a*_w_ = 0.2–0.8). Error bars represent the standard deviation in aspect ratio. (g) DSC scans of the matrix and nanocomposites previously equilibrated at different water activities (*a*_w_).

Enhancing gas barrier properties by adding nanoplatelets and sheets to polymer membranes is well established.^11,12,56^ We tested the influence of incorporating nanoplatelets with a high aspect ratio and surface area on the water vapor barrier properties. The MMT platelets significantly reduced the slope in water vapor sorption kinetics, particularly at low water activities (*a*_w_) (Figures 5a,b and S11). The impermeable filler’s high aspect ratio increased the tortuous path for water vapor molecules to pass through the composite. In addition, the nanocomposites show reduced water sorption capacity as the gelatin mass decreases, with an average drop of 62 ± 5% in 64MMT (Figure 5c). Up to *a*_w_ = 0.4, a further decrease in capacity can be attributed to the strong interactions between gelatin/MMT components, increasing hydrophobicity. However, adding platelets will also influence the free volume of the matrix polymer, increasing sample variation in water uptake (Table S1), especially in the case of gelatin, which can show supramolecular thermo-reversibility. In all samples, the equilibrium isotherms also show a clear step around *a*_w_ 0.6–0.8 (Figure 5c), possibly due to water plasticization, that is, increased segmental mobility and clustering phenomena.

By fitting the kinetic sorption curves (eq 9a), the Fickian diffusion parameter, ϕ_F_, also changes with water concentration (Figure 5d). We observe that the addition of MMT has shifted the non-Fickian transition from *a*_w_ = 0.4 to higher humidity (*a*_w_ = 0.6–0.8), a sign of anomalous diffusion that is loading-dependent. We determined the diffusion coefficients (*D*) of gelatin and nanocomposites (Figure 5e). The eq 9a fits agreed well with the experimental data (χ^2^ statistics, *p* < 0.05). In general, *D* increased with *a*_w_, from the hydrophilic gelatin content. The global decrease in *D* for nanocomposites with an increasing MMT content reflects the tortuosity hypothesis. This phenomenon should scale with the nanoplatelets’ concentration, orientation (⟨*P*_2_⟩), aspect ratio, and overlap factor. However, we find that the *D* in composites was higher than expected and did not follow the ⟨*P*_2_⟩ of high-aspect-ratio exfoliated nanoplatelets.

### Transport Aspect Ratio

**Figure 6:**
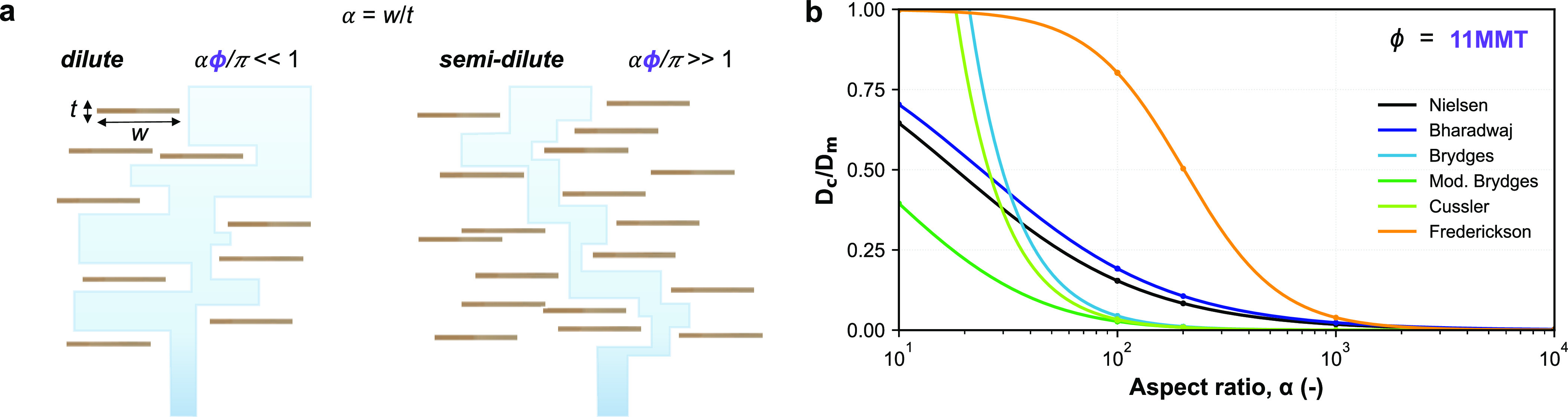
(a) Schematic depicting the tortuous path for gas diffusion through platelet-reinforced nanocomposites in the dilute (αϕ/π ≪ 1) and semidilute (αϕ/π ≫ 1) concentration regimes, where α and ϕ are, respectively, particle aspect ratio and volume fraction, as previously proposed by Fredrickson and Bicerano (1999).^57^ Particle aspect ratio, α, is defined by width, *w*, over thickness, *t*. Dilute regime: the platelet centers are spaced by a mean distance that exceeds their radius. Semidilute regime: the platelets overlap strongly imposing higher tortuosity. (b) Diffusion coefficient ratio between composite (*D*_c_) and matrix (*D*_m_) for a specific volume fraction, 11 vol % MMT, covering several orders of magnitude in α, as proposed by varying theoretical transport models.

The *effective* aspect ratio, α, of nanoplatelets was back-calculated from the diffusion coefficients using the modified Nielsen model proposed by Bharadwaj (2001), eq 11. This simple model considers the orientational order parameter ⟨*P*_2_⟩, which we obtained from WAXS results. The calculated α values started at the *a*_w_-averaged value of 185 nm (Figure 5f). However, for the higher MMT loadings (33 and 64MMT), we observed an apparent decline in α, which did not follow the expected trend in ⟨*P*_2_⟩. This is explained by contradictions with the assumptions made by the descriptive Nielsen’s model in eqs 10 and 11. Nielsen’s model assumes that platelets are monodisperse in size and perfectly overlapped (i.e., not stacked). Those were not the system conditions, and we expect random overlapping in a labyrinth effect, causing an α underestimation (Figure 6a). Moreover, the models assume a constant value of *D* for the matrix polymer. Curiously, the renaturation of gelatin supramolecular architectures has led to increased *D* values, likely from providing a “highway” for water diffusion (Figure S10). In our system, we have found that gelatin A presented different degrees of the triple helices with increasing MMT loadings (Figure 5f,g). Differential scanning calorimetry (DSC) also showed that the renaturation temperature of aggregates was dramatically influenced by the MMT filler at a determined water uptake (Figure 5g). For 64MMT, the matrix renaturation point is so much suppressed in the confined gel that it only shows a residual “melting” peak. On top of that, we imagine there might have been anomalous diffusion from interfacial phenomena at very high loadings (33–64MMT), as polymer confinement in the interphase can lead to properties different from the bulk, creating mobile water channels. This is substantiated by a possibly higher free volume in MMT-loaded samples, as shown by the degree of gelatin-based swelling (Table S1). Overall, we propose that the amorphous gelatin structure changes dramatically near a particle, be it a gelatin helix or a MMT platelet. These dynamic changes in matrix coefficient (*D*_m_) with increasing MMT incorporation prevent a very reliable α determination using the established theoretical framework. To circumvent this, future work should be done on measuring the barrier properties of nanocomposites for other gases such as O_2_, methanol vapor, and CO_2_, which plausibly are not very good plasticizers.

We further elaborate on the theoretical grounds for α determination via transport properties. It is important to restate that the effect of filler volume fraction has been somewhat described up to a semidilute regime (Figure 6a). The concentrated regime, including polymer confinement, edge extension, and (indirect) filler–filler interactions, is yet to be explored. In Figure 6b, we demonstrate how the adopted α model can dramatically influence the order of magnitude of the required diffusion ratios. Note the dramatic difference between the models, varying by almost 50% at the expected value for our studied MMT, α = 200 nm. Hence, the lack of comparative α models remains challenging in the filler-concentrated regime of (dynamic) polymer nanocomposites, certainly if we are to test the effects of structural order on properties.

We have demonstrated via mechanical and transport properties that a hydrogel affine deformation strategy can achieve continuous reinforcement and enhanced barrier properties through nanofiller immobilization and orientation. Future work shall extend this strategy to other compatible polymer nanocomposites by varying the individual components, shape, and aspect ratio of nanofillers, gelling, or network conditions (cross-linking) and exploring how to prevent nanoparticle wrinkling phenomena. In addition, special attention should be given to rheological studies in which the actual degree of affine deformation, network strength, and filler relaxation times might be determined. A key goal would be to design scalable hybrid systems that achieve close to perfect affine compression and very high levels of orientation, preferably at high loadings. In an ideal scenario, this would be combined with green processing for large-area fabrication of advanced bioinspired materials with consistent and reproducible properties.

## Conclusions and Perspectives

In this study, we propose that the orientation in many well-established waterborne nacre-mimetic methodologies cannot be achieved by 2D material assembly. Instead, it is often formed via a network that undergoes compressive affine deformation. This is based on the premise that any mean-field assembly process requires mobility to result in nanoparticle alignment, which is inevitably absent in cross-linked systems. We hereby investigate the affine network mechanism with a hydrogel system consisting of gelatin/MMT and solvent casting to produce nanocomposite films. The experimental design was set to establish comprehensive relationships among the alignment mechanism, structure, thermomechanical, and barrier properties as a function of filler loading, orientational order (⟨*P*_2_⟩ parameter), and aspect ratio.

Gelatin/MMT nacre-like composites were successfully fabricated under facile ambient conditions and formed dense lamellar structures up to exceptionally high MMT loadings, studied up to 64 vol %. First, the orientational order distribution in exfoliated nanocomposites was studied via WAXS (nanostructure) and electron microscopy (microstructure). Indeed, we find that the orientation distribution function of affine deformation, in contrast to the Maier–Saupe theory, is suitable for azimuthal intensity profiles in all nanocomposites. The affine deformation fwhm factor, representative of the compressive strain λ, allows us to estimate the solids concentration in which particle-immobilizing yield stress develops (1/λ). We find that the gelatin/MMT networks are, in reality, pseudo-affine since deformation does not occur until later in the evaporative process (1/λ_*z*_ > 1/λ_*z*__max_). Remarkably, for a wide range of nanoplatelet volume fractions, up to 64 vol %, we find an almost constant degree of compression (λ_*z*_) and respective order parameter ⟨*P*_2_⟩. This means a compatible hydrogel strategy is efficient in avoiding 2D material stacking at high loadings and can ensure an excellent degree of orientational order via immobilization (in our case, ⟨*P*_2_⟩_affine_ is scattered at about 0.7).

Next, we verified the influence of these exfoliated nanostructures, containing a high aspect ratio filler, on stiffness, increased heat resistance, and lower gas permeability. The constantly high orientational order is also reflected in the monotonic increase of reinforcement efficiency from modulus. Increased mechanics are kept even at very high RH (90%). The MMT aspect ratio, α, was further investigated via Halpin–Tsai composite theory. From the exfoliated state, we know that structurally, there must be a constant α—predicted around 200 nm—over the MMT volume fraction. However, Halpin–Tsai’s findings did not meet expectations, which we imagine might be due to platelet wrinkling. Thus, the ⟨*P*_2_⟩-based α values decreased with the filler concentration from 99 to 13 nm. For the same reason, the experimental modulus does not improve accordingly, with 23 GPa for 64MMT. The α parameter was also examined through water vapor diffusion. We observe a remarkable reduction in sorption kinetics and diffusion coefficients with an increase in MMT loading and diffusion tortuosity. However, for gelatin/MMT nanocomposites, we also observe that the matrix diffusion, *D*_m_, was altered by strong interactions with the clay component (reducing the level of gelatin renaturation). This also influenced the calculated α, estimated from the water diffusion data, which changed from about 200 to 60 nm. In addition, the models for estimating diffusion-based aspect ratios are yet to be translated for high volume fractions (high overlapping and labyrinth case), adding to underestimating this important morphological feature. Therefore, adjustments to the composite reinforcement and permeation theories are still needed to properly describe the high filler concentration regimes.

To conclude, we propose that the affine deformation mechanism is rather suitable to understand and indeed tailor nacre-inspired nanocomposites to a high level of orientational order and alignment, even at exceedingly high filler loading levels, which allows the development of advanced structure–property relationship-based optimized materials. The predictive power of the affine deformation model should allow for tuning of the degree of compressive strain (uniaxial drying) and associated alignment experienced by the entangled or immobilized 1D or 2D filler components. More importantly, because of the easy scalability potential of hydrogel affine deformation methods, this might broaden the scope for the practical application of advanced bioinspired nanocomposite materials.

## Methods

### Chemicals

Porcine skin gelatin (type A, 78–80 mM free COOH/100 g protein, 50,000–100,000 Da, gel strength 300, relative density 1.3 g cm^–3^), MgCl_2_, and KNO_3_ were purchased from Sigma-Aldrich and used as is. Sodium MMT (Na-MMT), CLOISITE-Na^+^, with *D*_50_ particle size <25 μm, basal spacing *d*_001_ of 11.7 Å, and density 2.86 g cm^–3^ was obtained from BYK Chemie GmbH, Germany, and used without purification or surface treatment. The platelet aspect ratio (length over thickness) of this dispersed Na-MMT was commonly reported within the range of 10–1000 nm but was typically 100–500 nm. The Na-MMT thickness was 1 nm. We have indirectly measured, via FIB-SEM, the average length of 212 ± 97 nm of dispersed nanoparticles in composite cross-section.^39^ All chemicals used were of analytical grade.

### Fabrication of Nacre-Inspired Nanocomposites

The fabrication of nacre-like gelatin/MMT composites was done according to our previously reported method.^39^ In short, a warm predispersed MMT suspension (3 w/v %) was added to a hot gelatin A solution (3 w/v %, 50 °C) according to the desired MMT volume fractions. Film-forming suspensions of protein/clay were prepared by vigorously mixing wet ratios at 70 °C. The pH of the stock and film forming solution was monitored. The dispersion was carefully poured into a polystyrene Petri dish and dried under ambient conditions to form a thin film.

The study aimed at a wide range of filler loadings ranging from 1 to 80 wt % Na-MMT, on a composite weight basis. In the solid state, this should be equivalent to up to 65 vol % Na-MMT. This was measured via TGA. Throughout this study, the composite clay content is expressed as a volume percentage (vol %). The samples are denoted as *X*MMT, where *X* is the volumetric MMT fraction percentage in the composite.

For the following characterization analyses, the films were additionally conditioned for 1 week in a desiccator containing silica gel at room temperature. This step was necessary to ensure there was an absence of freely bound water as the gelatin polymer has a hydrophilic nature.

### Characterization

#### Thermogravimetric Analysis

The nanocomposite samples were cut into snippets and analyzed using a thermogravimetric analyzer (TGA 8000, PerkinElmer, USA) from 30 to 900 °C using a heating rate of 10 °C min^–1^. The sample weight was in the range of 4–6 mg. Corundum crucibles were used, and air was used as a purge gas at a flow rate of 20 mL min^–1^. The method included isothermal steps at 105 and 900 °C for water removal and final weight equilibration, respectively. Due to the usage of an oxidative environment and the negligible ash content of gelatin, the final residue represents the weight percentage of MMT. These concentrations were later converted into vol % MMT, ranging from 0.4 to 64%.

#### Fourier-Transform Infrared Spectroscopy (FTIR)

FTIR spectra were recorded using a Nicolet 6700 (Thermo Fisher Inc., USA) spectrometer with a frequency range from 4000 to 600 cm^–1^, at a resolution of 2 cm^–1^. The acquired spectrum of dry films was an average of 128 scans.

#### Flame Resistance Test

The resistance of films to flame exposure using a (propane/butane) blue oxidizing torch was tested. When possible, the samples were exposed to the flame from both sides of the film until ignition or material radiation emission was observed (up to 30 s). SEM images were also taken of the burned specimens. Prior to flame tests of the films, the dried films were conditioned for 2 days at 50% RH and RT.

#### Scanning Electron Microscopy

The cryo-fractured surface of the film samples was imaged with a JSM-6010LA JEOL (JEOL Ltd., Tokyo, Japan) SEM at an accelerating voltage of 8 kV and a close working distance (7–9 mm). Measurements and image transform analysis of the micrographs were performed by Gwyddion software (1D FFT filter).

#### Wide-Angle X-ray Scattering

WAXS was performed using a Bruker AXS D8 Discover with a VÅNTEC 2D detector and Cu Kα radiation (λ = 1.54184 Å) at 50 kV and 1 mA. A point collimator of 0.3 mm was used, and the sample to detector distance was 30 cm parallel (incident beam at a glancing angle) and perpendicular to the film surface. For basic interpretation and data curation of the X-ray diffraction, Bruker software (DiffracSuite.EVA version 5.1, Bruker, USA) was used. From the obtained 2D-XRD results, the radial integrations and particle dispersion analysis thereof can be found at Espíndola et al. (2023).^39^

#### Orientation Distribution Functions and ⟨*P*_2_⟩ Order Parameter

The alignment in nacre-like nanocomposites was studied by means of orientation distribution functions. Applying orientation models is useful to understand which alignment mechanisms might be in place. The following functions were fitted to the WAXS azimuthal intensity profile data at the maximum intensity region and are well-established for quantifying the orientation order parameter ⟨*P*_2_⟩.

#### Maier–Saupe Model

The Maier–Saupe model describes the long-range contributions of a nematic environment on the orientation of a single particle along one direction.^35,36^ Thus, the model is often used in liquid crystal theory in the self-assembly of nematic polymeric crystallites. Equation 1 describes the shape of the ODF resulting from this model
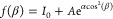
1where *I*_0_ is the intensity baseline, *A* is a normalization constant, α is the width of the curve and is directly related to the order parameter ⟨*P*_2_⟩, and β is the azimuthal angle.

#### Affine Deformation Model

The affine deformation model, originally coming from the field of ideal rubber elongation,^38^ has been modified to describe orientational order in liquid crystals and composite systems.^24,32,58,59^ In an affine deformation, due to cross-linking and the development of yield stress, local deformations are equal to global deformations. Therefore, with the model, external deformation forces such as shrinkage can be linked to the extent of alignment. Using the modified model, one finds that the orientational order can be developed from particle immobilization, via a (pseudo)network formation and subsequent vertical consolidation over drying. Equation 2 describes the ODF predicted by this model
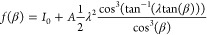
2where *I*_0_ is the intensity baseline, *A* is a normalization constant, λ is a degree of (vertical) compression and is directly related to the order parameter ⟨*P*_2_⟩, and β is the azimuthal angle, which corresponds to the platelet angle with respect to the *z*-axis.

#### ⟨*P*_2_⟩ Order Parameter

The sample degree of orientation derives from the fitted orientation models by calculating the order parameter ⟨*P*_2_⟩, using eq 3
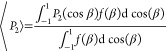
3where *P*_2_(cos β) is a second-order Legendre polynomial of cos(β): . Analytical solutions for the ⟨*P*_2_⟩ calculation have also been previously demonstrated.^60^ The fit-optimized values of width α and degree of consolidation λ were used for Maier–Saupe and affine deformation regression functions, respectively. For randomly oriented samples ⟨*P*_2_⟩ = 0, while for complete anisotropy ⟨*P*_2_⟩ = 1. For a liquid crystal nematic phase, ⟨*P*_2_⟩ typical values lie between 0.3 and 0.7.

The orientation functions were fitted to the experimental data by running code in Python language software and modules Scipy and Numpy. Integrals were calculated using the scipy.integral.quad built-in function. The regression goodness-of-fit for orientation models was evaluated with reduced chi-squared statistics, where Efron’s pseudo-*R*^2^ coefficient of regression is reported.

#### Dynamic Mechanical Thermal Analysis

DMTA was performed on a PerkinElmer DMA-7e instrument (PerkinElmer, USA). DMTA experiments were performed in a tensile mode at a frequency of 1 Hz from −50 to 300 °C temperature range at a heat rate of 5 °C min^–1^, with film dimensions of roughly 10.0 × 3.0 × 0.15 mm. The thickness of the films was measured with the aid of a digital micrometer. Prior to this analysis, the films were extensively dried for 1 day at 40 °C.

DMA was also performed at a constant temperature (30 °C) but with varying RH using a DMA Q800 (TA Instruments, USA) with a controlled humidity chamber accessory (HumiSys, InstruQuest Inc., USA). This allowed us to monitor the moduli of films at varying humidity or water activity, *a*_w_ = 0.01, 0.20, 0.40, and 0.60 to 0.90.

To better understand the influence of nanoplatelet addition to mechanics, we used a semiempiric composite theory, taking into consideration the individual contributions of matrix and filler (⟨*P*_2_⟩ order parameter, aspect ratio, volume fraction, and modulus). The Halpin–Tsai model is widely used to estimate the reinforcement effect of filler in polymer clay composites (Halpin and Kardos (1976)),^61^ which can be written in the following closed form
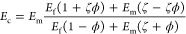
4where *E*_c_, *E*_m_, and *E*_f_ are the moduli values of the nanocomposite, matrix, and filler, respectively, and ϕ and ζ are the volume fraction and shape factor of the filler. The latter depends on filler geometry, orientation, and aspect ratio (α). The value for *E*_f_ is hereby assumed to be 172 GPa for a perfect mica crystal, based on Shell and Ivey (1989).^62^ Since gelatin renaturation was found to be decreasing with filler incorporation, we assumed an *E*_m_ value based on a solvent-casted amorphous film (4 GPa).

Based on the filler shape factor, ζ, this model reduces to series or parallel contributions to modulus, which can be used as boundary conditions for real application composites.^47^ If the shape factor and aspect ratio are much lower than the modulus ratio of the filler and polymer matrix (ζ ≪ *E*_c_/*E*_m_), the model converges to the series model
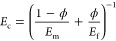
5

In the opposite case, the filler shape factor and aspect ratio are much higher than the modulus of the filler and the polymer matrix (ζ ≫ *E*_c_/*E*_m_), the Halpin–Tsai model converges to the parallel version
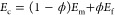
6

Using eq 4, we can also calculate the effective modulus for composites containing perfectly anisotropic and isotropic platelets. Furthermore, by incorporating the previously obtained order parameter ⟨*P*_2_⟩ values from WAXS, we can correct the composite modulus based on orientation applying eq 7(63)
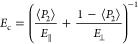
7where *E*_∥_ and *E*_⊥_ are tensile moduli values of the composite in the radial direction of the platelets (unidirectional) and perpendicular to the platelets (random), respectively. For the moduli components, Van Es et al. (2001)^64^ has previously derived the shape factors, ζ, for *E*_∥_ and *E*_⊥_ to be equal to 2/3α and 2, respectively.

#### Dynamic Water Vapor Sorption and Kinetic Model

The effect of nanoplatelet incorporation on water vapor capacity and kinetics was studied by dynamic water vapor sorption (DVS). DVS was performed in a TA Instruments Q5000 SA instrument (TA Instruments, USA) by measuring the increment in mass from the water vapor of the gelatin/MMT films or ground powder. The isotherms were collected by employing a method with 4 steps by varying the water activity (*a*_w_) from equilibrated 0.01 to 0.20, 0.40, 0.60, and 0.80. Humidity was maintained in the sample environmental chamber by a laminar flow with a wet-dry vapor mixing at a constant flow rate with feedback control. The time at each *a*_w_ was iterated until a mass plateau was reached for most samples.

To interpret the water sorption kinetics, we use a Crank derivation to calculate the diffusion coefficient, assuming complete Fickian behavior, infinite sheet geometry, and a constant initial concentration throughout the sample and surface^65^
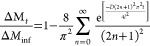
8where Δ*M*_*t*_ and Δ*M*_inf_ represent the water mass uptake at time *t* and at equilibrium, respectively. *D* is the effective diffusion coefficient over a specific concentration interval and *l* is the half thickness of the film.

Nevertheless, there is a lag phase before Fickian diffusion takes place in almost all samples, as visualized by a curvature convex to the time axis before the actual Fickian regime. This lag is related to the time necessary for attaining equilibrium saturation at the polymer/water–vapor interface (τ_S_), which is mostly reported to be a step from an instrumental anomaly and not a material property.^66^ Hence, to calculate the diffusion coefficient from these sigmoidal sorption curves, we applied exponential time-dependent boundary conditions, including τ_S_, derived by Long and Richman (1960).^67^ In addition, anomalous non-Fickian relaxations are also possible within the gelatin/MMT system due to morphological changes. Hence, the used solution of a time-dependent term coupled to the Fickian expression is given by the expressions 9a-b, with a non-Fickian term as proposed by Berens and Hopfenberg (1978).^66^ The original Fickian term is recovered if τ_S_ = 0 and τ_R_ tends to ∞
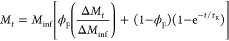
9a
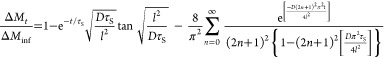
9bwhere Δ*M*_*t*_ and Δ*M*_inf_ represent the water mass uptake at time *t* and at equilibrium, respectively. *D* is the effective diffusion coefficient and *l* is the half thickness of the film. τ_S_ is the characteristic time for attaining saturation at the polymer/water–vapor interface, which is mostly reported to be a step from an instrumental anomaly and not a material property. The time τ_R_ is related to non-Fickian relaxations and is separated from the Fickian phenomena by the weighing factor ϕ_F_. Curve fitting was done in Python language software and the initial boundaries for the *D* parameter were set to match the Fickian slope in the linear region (normalized mass uptake over *t*^0.5^).

A reduction in diffusion coefficient is attributed to the tortuous path imposed to the penetrating water vapor by the incorporation of high aspect ratio platelets.^68,69^ Hence, it can be directly linked to the nanostructure in composites and the effective filler aspect ratio. A few models have been in use to describe the effects of incorporating impenetrable platelets to diffusion, based on concentration, aspect ratio, overlapping, and order.^63^ Nielsen (1967)^68^ defined a simple model by assuming perfectly oriented and overlapped monodisperse platelets
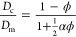
10where *D*_c_ and *D*_m_ represent, respectively, the diffusion coefficient of composite and matrix, α is the width to thickness aspect ratio, and ϕ is the volume fraction of platelets.

To include the effect of orientational order on diffusion, via the order parameter, we use the modified Nielsen model proposed by Bharadwaj (2003)^69^
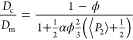
11where *D*_c_ and *D*_m_ represent, respectively, the diffusion coefficient of composite and matrix, α is the width to thickness aspect ratio, ϕ is the volume fraction of platelets, and ⟨*P*_2_⟩ is the composite order parameter, for example, calculated from affine deformation ODF.

In addition, other descriptive models were investigated, that is, Cussler,^70^ Fredrickson,^57^ Brydges,^71^ and modified Brydges.^56^

#### Differential Scanning Calorimetry

DSC experiments were performed to characterize the thermal behavior of nanocomposites. First, film snippets were acclimated to different RH environments (0, 33, 93% RH) using silica gel and saturated salt solutions (MgCl_2_ and KNO_3_). The water uptake capacity at the end of 2 weeks was also measured gravimetrically. The DSC method consisted of heating the sample from −50 to 150 °C, at a rate of 3 °C min^–1^, on a PerkinElmer Diamond instrument with two 1 g furnaces calibrated with indium. Nitrogen gas was used to purge the thermal analyzer at a flow rate of 50 mL min^–1^. Stainless steel pans with O-ring seals were used for hermetically encapsulating the equilibrated samples (20 mg). An identical empty reference pan was used. The pans were sealed according to supplier instructions (PerkinElmer). Data visualization was carried out by the Python script, in which the *y*-axis refers to endothermic transitions. Each thermogram was analyzed for the melting (or denaturation) event.

## Supporting Information Available

Interpretation of nanoplatelet azimuthal X-ray diffraction analysis; TGA; frames WAXS diffraction; affine deformation of renatured gelatin analysis WAXS and SEM; ODF fits with Gaussian convolution; affine deformation in lateral direction; DMTA; DMA under varying RH; DVS of amorphous and renatured gelatin; DVS nanocomposites; and flame resistance test (PDF)
