# Supplementary material for: Affine Deformation and Self-Assembly Alignment in Hydrogel Nanocomposites
Source: Macromolecules. 2023 Nov 22;56(23):9839–52. doi: 10.1021/acs.macromol.3c01638 (PMC10720479; doi:10.1021/acs.macromol.3c01638)
Supplement: Supplementary file 1 — ma3c01638_si_001.pdf [file ma3c01638_si_001.pdf]

Supporting information

Affine deformation and self-assembly alignment in hydrogel nanocomposites

Suellen Pereira Espíndola[†,], Ben Norder[†], Kaspar M. B. Jansen[‡], Jure Zlopasa[¶], Stephen J. Picken[†,*]*

† Department of Chemical Engineering, Faculty of Applied Sciences, Delft University of Technology, Van der Maasweg 9, 2629 HZ Delft, The Netherlands

‡ Department of Sustainable Design Engineering, Industrial Design Engineering, Delft University of Technology, Landbergstraat 15, 2628 CE Delft, The Netherlands

¶ Department of Biotechnology, Faculty of Applied Sciences, Delft University of Technology, Van der Maasweg 9, 2629 HZ Delft, The Netherlands

*E-mail: S.PereiraEspindola-1@tudelft.nl.

*E-mail: S.J.Picken@tudelft.nl.

Contents

Supporting Texts *3*
Supporting Text S1. Interpretation of nanoplatelet azimuthal X-ray diffraction analysis **3**
Supporting Text S2. General discussion on compatibility of the gelatin/MMT system **6**
Supporting Figures and Tables *8*
TGA **8**
WAXS frames **9**
Affine deformation of renatured gelatin **10**
ODF Gaussian convolution **11**
Affine deformation in lateral direction **12**
DMTA **13**
DMA under varying RH **14**
DVS amorphous and renatured gelatin **15**
DVS nanocomposites **16**
Film swelling **17**
Flame resistance test **18**

Supporting Texts

Supporting Text S1. Interpretation of nanoplatelet azimuthal X-ray diffraction analysis

We wish to examine the orientational order of nanoplatelets (or nanosheets) within composite films *via* azimuthal scans of X-ray scattering features (beam parallel to sample at a glancing angle). These resulting features have intensity perpendicular to the particle xy-plane. Figure S1 illustrates how the azimuthal X-ray scattering of a perfectly oriented nanoplatelet should appear, assuming different scenarios.

A hypothetical isolated infinite diameter, infinitely thin, and perfectly oriented platelet (or sheet) will exhibit scattering along the normal of the film xy-plane. Let's call this the scattering beam, as it is an infinitely thin straight line of intensity without any lateral spreading for increasing scattering vector, q. So, it is a ray of a 1D delta function with $I(q) = I(0) \rightarrow \infty$.

In practice, however, the platelet is not infinitely thin, and may have an internal structure, *e.g.* the Tetrahedral-Octahedral-Tetrahedral (T-O-T) triple layer constituting a MMT nanoclay platelet. This gives rise to some intensity variations along the beam.

On top of that, the width of the beam will be finite due to: (1) finite diameter of the MMT film, (2) possible internal defects and grain boundaries of the T-O-T layers, (3) possible loss of correlation due to thermal stress fluctuations (random bombardment of molecules in the order of k_BT), and (4) possible particle wrinkling undulations or structurally related defects. Effects 1 to 3 give rise to the delta function beam to spread out laterally to a 'searchlight' beam with a radial intensity distribution. In view of the multiple effects in (1 – 3) it is safe to assume a Gaussian-type intensity variation of the beam. Effect (4) causes some azimuthal disorder as the wrinkles are tilted out of plane, so the Gaussian beam distribution will diverge out to a (thermal or roughness) Gaussian distribution along the azimuthal circle. The result of this is that a perfectly oriented real MMT platelet (ensemble of isolated MMT platelets) will have a

scattering beam closer to a ‘wedge’. Therefore, we have a slight azimuthally and radially spread X-ray beam of intensity perpendicular to the MMT platelet orientation.

Effect on calculated $<P_2>$

If we have additionally some $<P_2>$ order parameter and associated azimuthal orientation distribution function, the azimuthal arcs will cause a blurry wedge-shaped intensity normal to the symmetry axis of the orientational order (we will assume that is still perfectly aligned along the film normal, no collective tilt of the nanoplatelet is allowed).

A measured azimuthal intensity will then reflect the orientational order of the MMT platelet distribution function however there will be broadening of the peak due to effect (4) (independent of scattering vector $|q|$), and from effects (1 – 3) a $|q|$ dependent broadening due to the Gaussian width of the ‘searchlight’ beam. These broadening effects are convoluted with the *e.g.* affine ODF and can be eliminated by measuring the azimuthal ODF at multiple $|q|$ ranges. In many cases, effect (4) can be assumed to be negligible, but it would give rise to $<P_2>$-experiment = $<P_2>$-wrinkle*$<P_2>$-affine. The $<P_2>$-wrinkle factor should be close to 1, maybe 0.97-0.99.

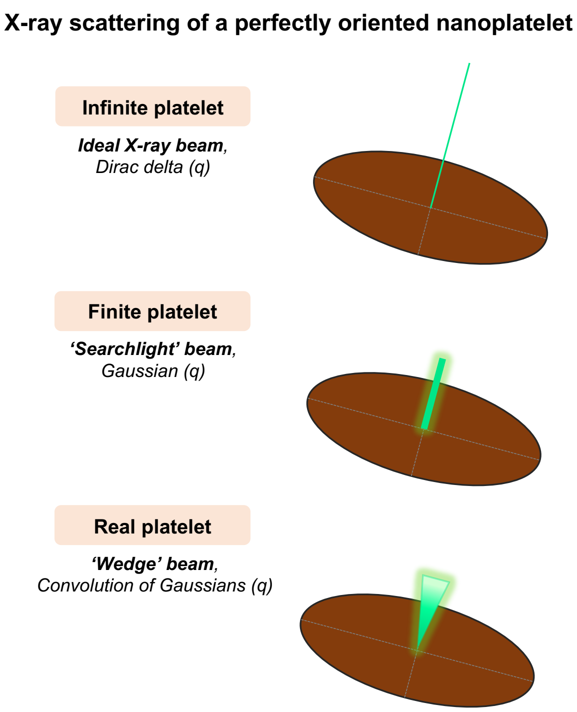

Figure S1. The proposed X-ray scattering of a perfectly oriented nanoplatelet considering an infinite platelet, finite platelet, and real platelet.

Supporting Text S2. General discussion on compatibility of the gelatin/MMT system

The gelatin-MMT specific interactions will affect the extent of nanoplatelet interfacial adhesion, gelation, and yield stress developed during affine deformation. Such interactions are clearly dependent on MMT fractions and pH conditions. Gelatin is an amphoteric protein; therefore, pH fluctuations will change its total net charge and functional group interactions. At pH < pI, gelatin insertion probably happened with cation replacement (Na^+ in clay interspacing to $-NH_3^+$ group from matrix)[50,69]. Under this state, electrostatic forces between gelatin-MMT were favored, with building blocks of clay and protein shell arrangements being formed. This strong attraction in combination with repulsion among gelatin macromolecules most likely resulted in more extended chain conformations and interactions. At exactly pH = pI, gelatin has neutral net charge, so intercalation is less favorable than in the previous case due to higher gelatin-gelatin hydrophobic interactions, i.e., lower gelatin solubility, and so entropic contributions become more relevant. The sample 64MMT was surely at pH=pI and, however decreased, the MMT basal spacing still suggests polymer insertion[39], therefore, there was an effective core-shell formation. It is possible that the high charge in MMT platelets can polarize the amphoteric gelatin even when the individual charges are equal. In addition, we propose the high clay concentration and protein-clay interactions in solution do not allow for significant coil to helix tridimensional transition of gelatin chains as the suspension cools down. Although the confinement of the polymer chains inside the silicate galleries results in a decrease in the overall entropy of the chains/helices, this penalty may be compensated by the increase in the freedom of the hydrated Na^+. Therefore, the total entropy of the intercalation should become negative, what is beneficial to exfoliation. In addition to supramolecular interactions, the gelatin/MMT system dynamics also must have been favoring exfoliation due to entropic repulsion of (coated) clay platelets.

An enhancement in mechanical, thermal, and barrier properties deriving from oriented MMT loaded on gelatin (bovine, porcine, teleostean) have already been previously reported for composite materials. Most studies so far have focused on low filler content (< 17 wt.% or ~8 vol.% MMT) and report on an increase in heat distortion temperature and lower water and gas permeability[17,44,70]. Applications such as absorbents, rheology modifiers, antistatic, and flame-retardant materials are observed in such polymer clay nanocomposites[17]. However, how such properties can be systematically tuned by exploring its actual orientation mechanism, *i.e.*, affine deformation, were not previously investigated. For that, careful studies over the impact of the type of immobilization mechanism (type of gelation and time it develops) would be useful; for instance, how it can be changed by temperature (with respect to the gelatin renaturation temperature), pH, solvent polarity, and dielectric medium constant, as these factors would affect the gelatin and gelatin-MMT junction interactions.

Supporting Figures and Tables

TGA

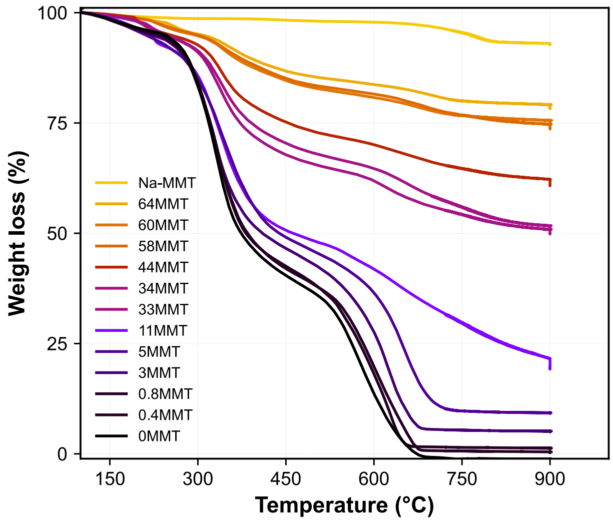

Figure S2. TGA scans of all gelatin and gelatin/MMT samples.

WAXS frames

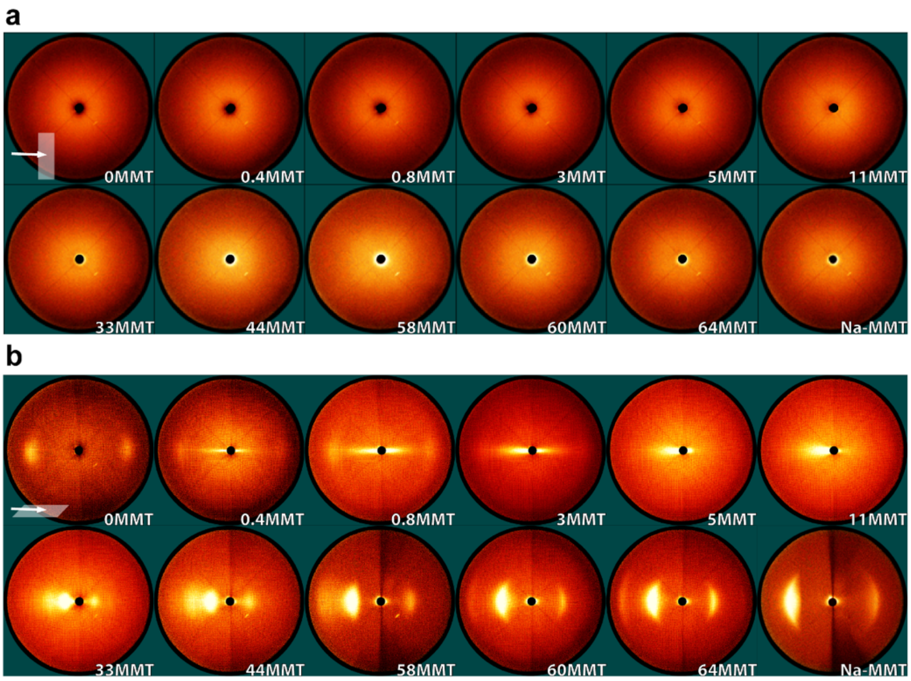

Figure S3. 2D transmission X-ray scattering images of gelatin and gelatin/MMT samples with varying MMT content at beam inclination (a) perpendicular and (b) parallel (at a glancing angle) to the plane of the films. The scattering patterns in (b) reveal anisotropic peaks (reproduced with permission from Espíndola et al. (2023)[39]).

Affine deformation of renatured gelatin

Gelatin renaturation of collagen-like triple helices has been observed via X-ray scattering (WAXS) and scanning electron microscopy (SEM) (Figure S4). Gelatin consists of single or multi-stranded polypeptides, with possible proline helix conformations. The coil renaturation is expected to appear with the formation of a helical secondary structure and subsequent aggregation of the helix joints. In this, the network appears from H-bonding of intra- or inter-chain interactions between hydroxyl and carbonyl groups. The aggregates form a fibrous network which can uniaxially deform during evaporation, consolidating into an anisotropic gel.

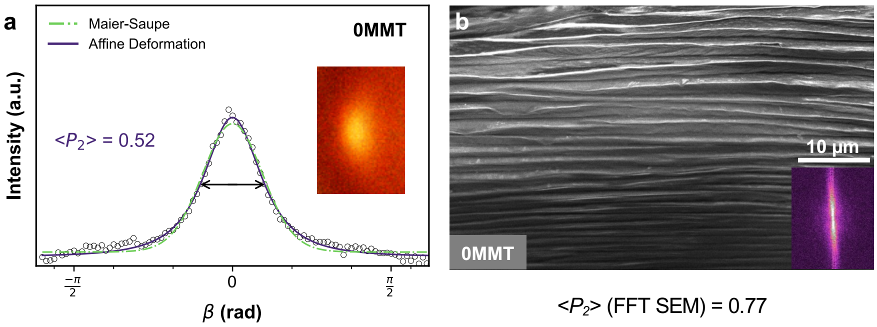

Figure S4. (a) WAXS feature, azimuthal intensity profile, and calculated orientation distribution functions of affine deformation (dark purple solid lines) and Maier-Saupe (light green dashed lines) for renatured gelatin A film (helical aggregates). The renaturation is observed by the appearance of equatorial diffraction arcs. (b) SEM cross-section image of gelatin A film (0MMT), where inset shows corresponding fast Fourier transforms (FFT) of the image for peak integration. The $\langle P_2 \rangle$ values depicted in images were determined via affine deformation ODF.

ODF Gaussian convolution

Gaussian Convolution

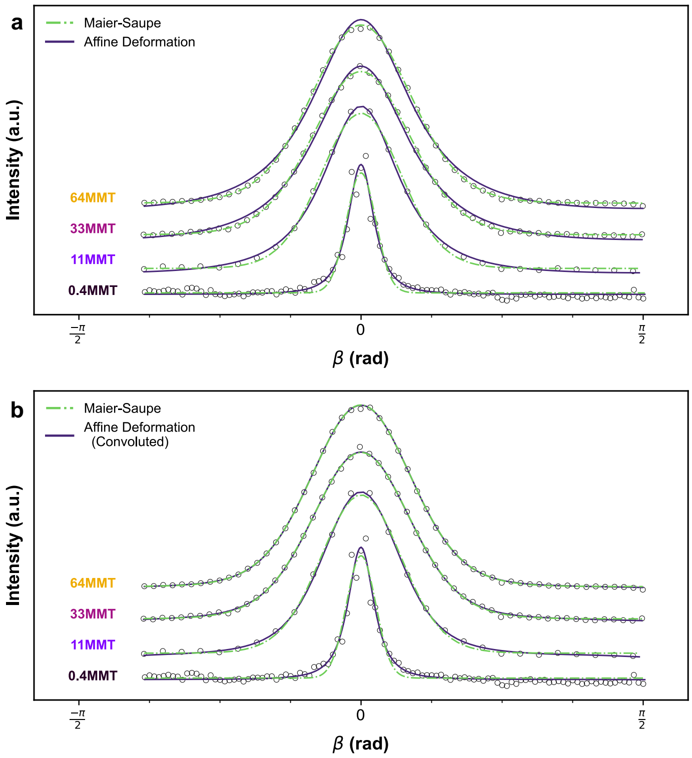

Figure S5. Azimuthal intensity profiles corresponding to the d_{001} reflection at different MMT volumetric loadings. The lines are the curve fit results for the Maier-Saupe (light green) and affine deformation (dark purple) models convoluted to a Gaussian function.

Affine deformation in lateral direction

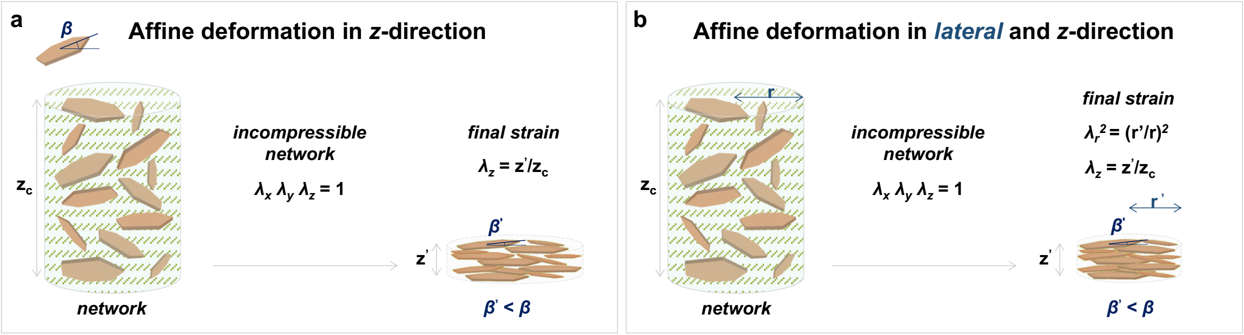

Figure S6. Schematic illustration of (a) affine deformation in the vertical z-direction for gelatin/MMT; (b) affine deformation in both the lateral (or radial) and vertical z-direction for gelatin/MMT. The rubber elasticity theory postulates that the network is incompressible, thus, the lateral and vertical strains can be correlated through $\lambda_x\lambda_y\lambda_z = \lambda_r^2\lambda_z = 1$.

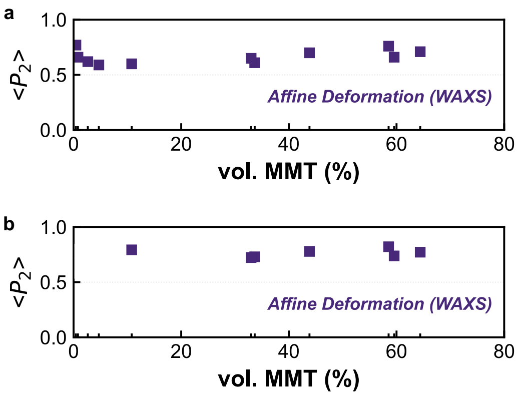

Figure S7. Order parameter $<P_2>$ dependence on MMT volume fraction: (a) accounting only for vertical compression λ_z; (b) accounting for vertical compression and this correcting factor by the degree of lateral shrinkage (λ_r^2).

DMTA

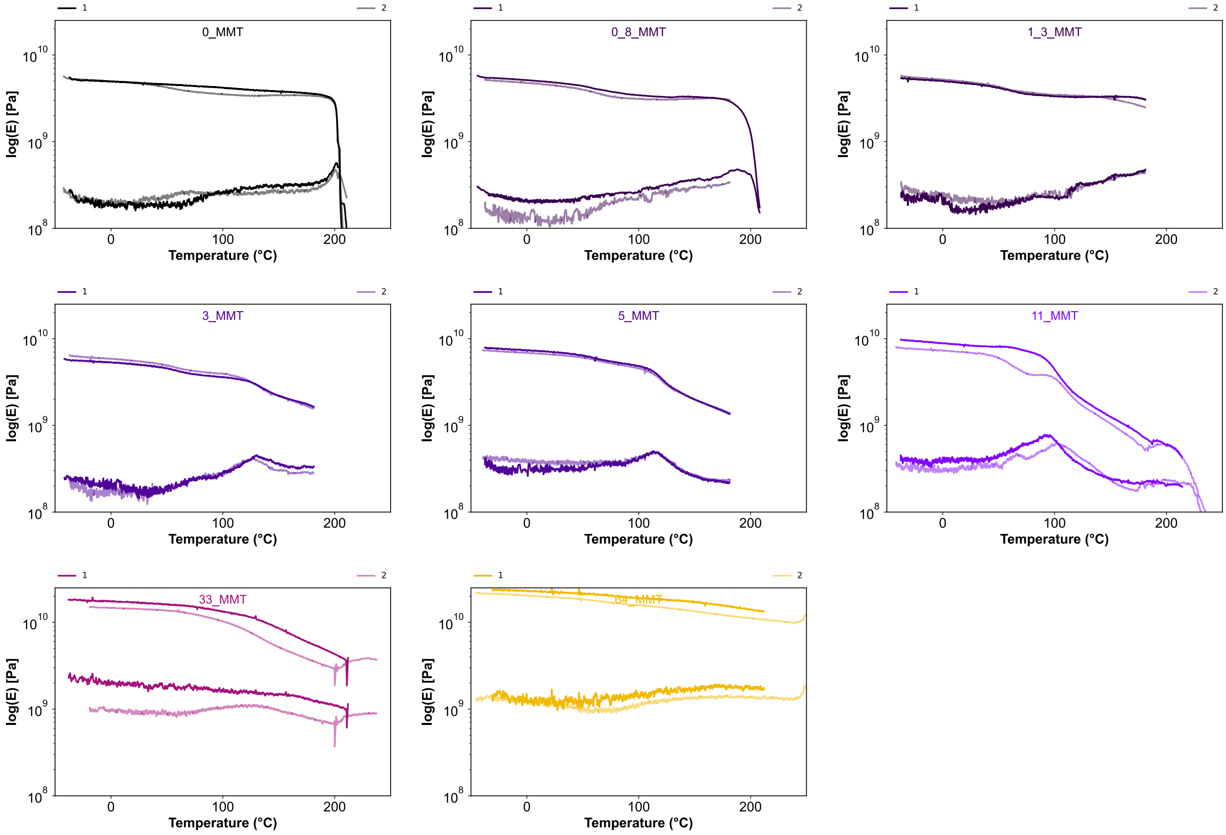

Figure S8. Full DMTA data of dry gelatin/MMT nanocomposites with increasing MMT loadings.

DMA under varying RH

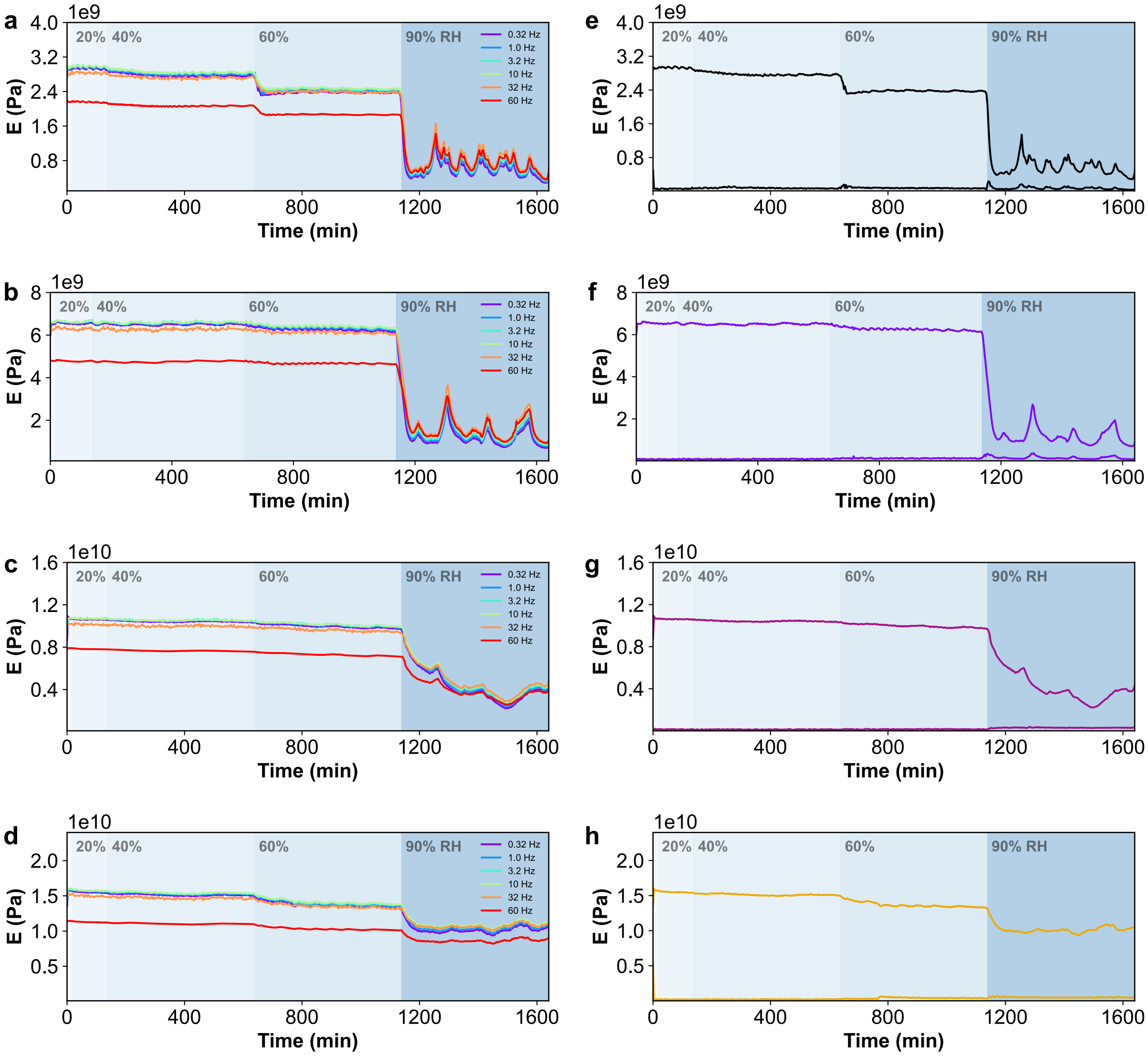

Figure S9. DMA analysis at 30 °C and different relative humidity ranges for gelatin/MMT nanocomposites at multiple frequencies and increasing MMT loadings (left column) and at 1 Hz and varying MMT loadings (right column). The composite data is displayed from top to bottom as: 0MMT (a, e); 11MMT (b,f); 33MMT (c, g); 64MMT (d, h).

DVS amorphous and renatured gelatin

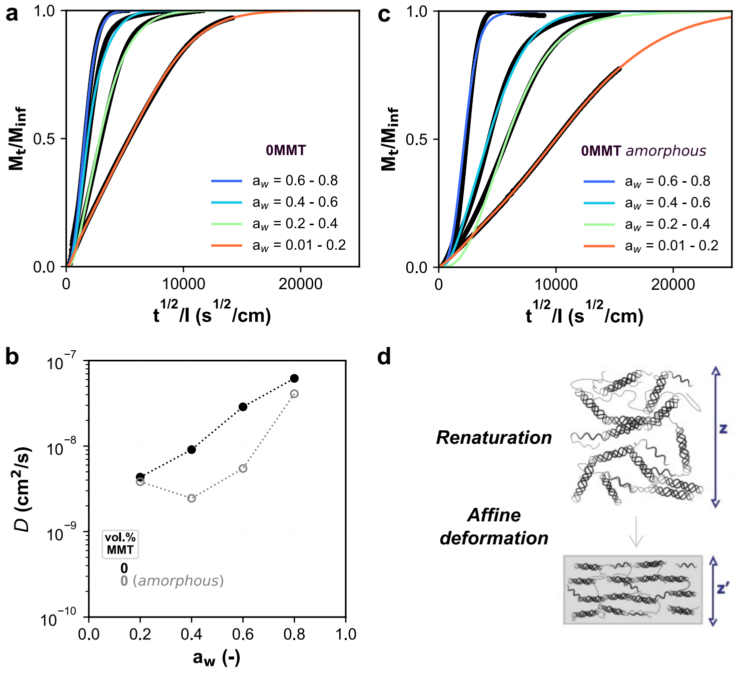

Figure S10. Kinetic water sorption of renatured gelatin (a) and amorphous gelatin (c) at 30 °C. (b) Water diffusion coefficient, *D*, calculated with eq 9 from the kinetic sorption curves for the different gelatins. (d) Schematic illustration of renaturation and affine deformation of renatured triple helix aggregated domains, creating a different microstructure.

DVS nanocomposites

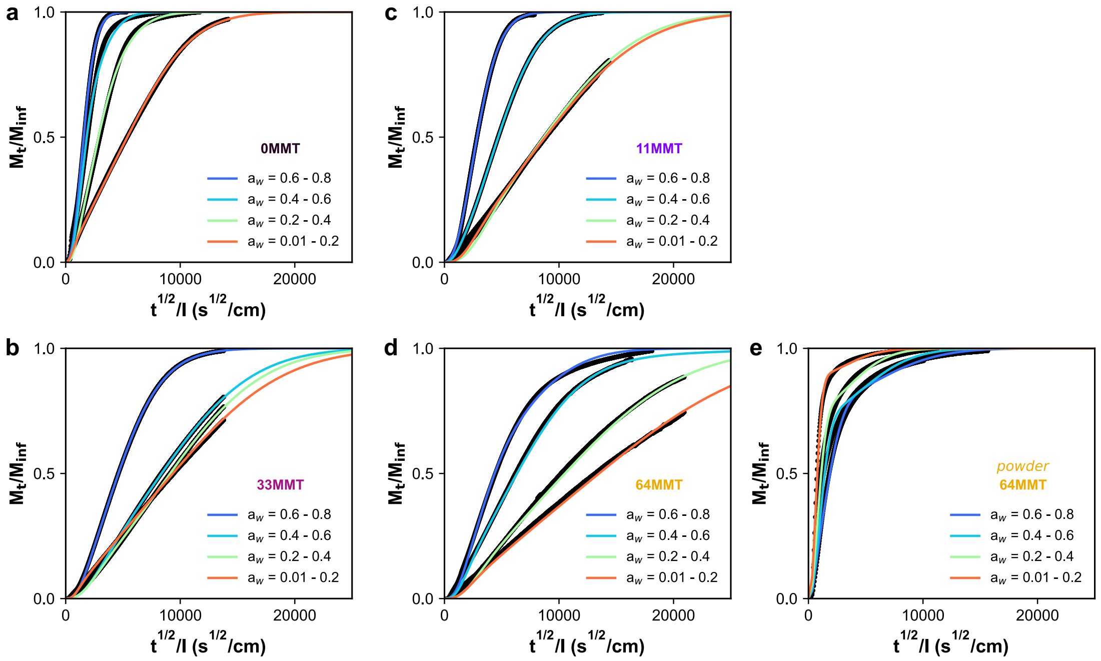

Figure S11. Kinetic water sorption of gelatin and gelatin/MMT nanocomposites at 30 °C. In (e), the composite 64MMT was first pulverized and ground to a fine grain size.

Film swelling

The gelatin polymer has a hydrophilic nature and the thickness of film snippets had to be corrected by water-induced swelling. From the large surface area to volume, we assume that film snippet swelling is one-dimensional. The thickness at a certain relative humidity, d_{RH}, is calculated as follows:

$$\frac{1}{d_{RH}} = \frac{1}{d_{dry}}(1 - \phi_{H_2O}) \qquad \text{(eq S1)}$$

where d_{RH} is the film thickness corrected for sample swelling by water vapor intake, d_{dry} is the measured thickness of dry films, and ϕ_{H2O} is the equilibrium water uptake of the composite.

Table S1. Thickness of gelatin/MMT film snippets under dry and humid conditions and the calculated gelatin swelling ratio

Sample	Dry thickness, d_{dry} (µm)	Expected humid thickness, d_{RH}, from eq S1 (µm)	Measured humid thickness, d_{RH} (µm)	Gelatin swelling ratio, SR/ϕ_{gel}
0MMT	169±4	220±7	190±5	12%
11MMT	167±5	201±6	205±6	26%
33MMT	184±9	209±10	204±10	17%
64MMT	114±5	125±5	122±5	21%

d_{dry}: measured dry composite thickness; d_{RH}: calculated or measured humid composite thickness; SR: composite swelling ratio (w_{humid}–w_{dry} / w_{dry}); ϕ_{gel}: gelatin volume fraction on composite basis.

Flame resistance test

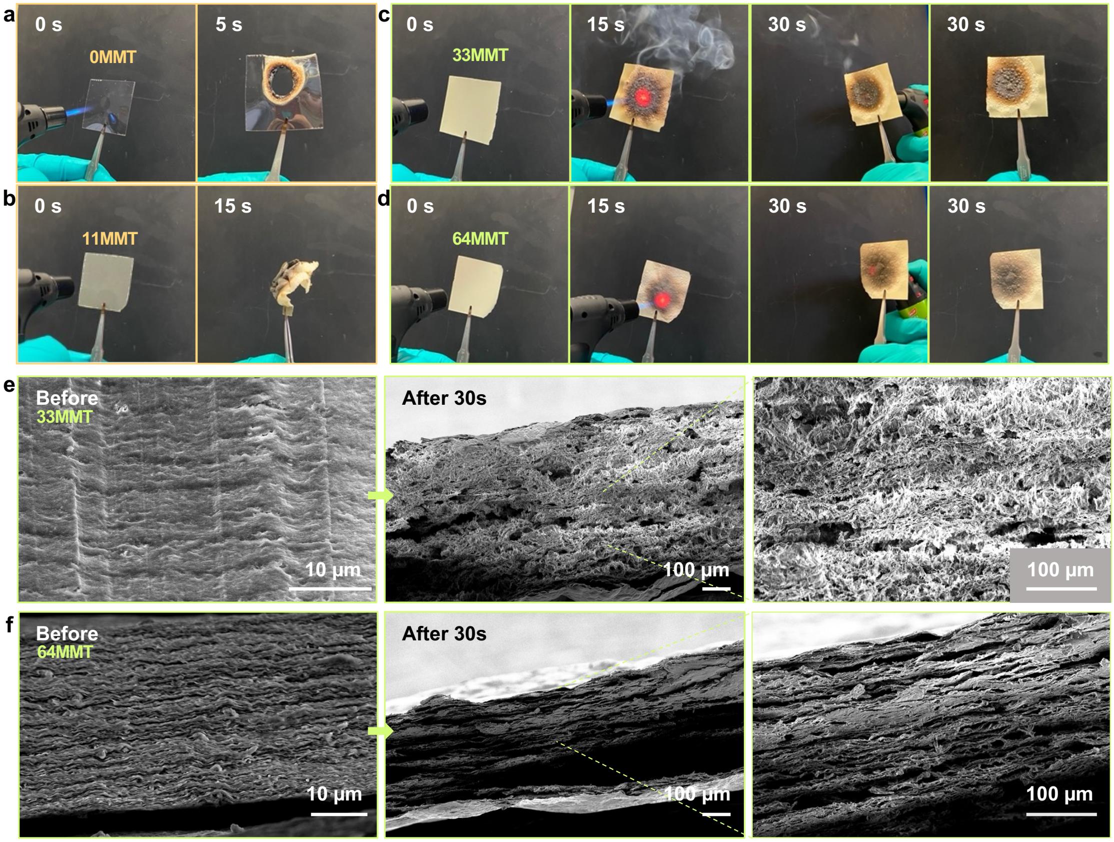

Figure S12. Flame resistance tests of gelatin and gelatin/MMT nanocomposites: 0 MMT (a), 11MMT (b), 33MMT (c), 64MMT (d). The corresponding SEM micrographs for 33MMT (e) and 64MMT (f) before and after flame exposure.
